# Anti-Obesity and Gut Microbiota Modulation Effect of Secoiridoid-Enriched Extract from *Fraxinus mandshurica* Seeds on High-Fat Diet-Fed Mice

**DOI:** 10.3390/molecules25174001

**Published:** 2020-09-02

**Authors:** Sen Guo, Haoan Zhao, Zhongxiao Ma, Shanshan Zhang, Mingrou Li, Zhaojing Zheng, Xiameng Ren, Chi-Tang Ho, Naisheng Bai

**Affiliations:** 1 College of Food Science and Technology, Northwest University, 229 Taibai North Road, Xi’an 710069, China; guosen0828@163.com (S.G.); haoan_zhao@126.com (H.Z.); 201921137@stumail.nwu.edu.cn (M.L.); zhengzhj@nwu.edu.cn (Z.Z.); renxiameng@stumail.nwu.edu.cn (X.R.); 2 Department of Pharmaceutical Engineering, College of Chemical Engineering, Northwest University, 229 Taibai North Road, Xi’an 710069, China; zhangss@stumail.nwu.edu.cn; 3 College of Chemical Engineering, Northwest University, 229 Taibai North Road, Xi’an 710069, China; mazhongxiao@stumail.nwu.edu.cn; 4 Department of Food Science, Rutgers University, 65 Dudley Road, New Brunswick, NJ 08901, USA

**Keywords:** *Fraxinus mandshurica* seed, secoiridoids, anti-obesity, gut microbiota, pancreatic lipase

## Abstract

Previously we conducted a phytochemical study on the seeds of *Fraxinus excelsior* and isolated nine secoiridoid compounds with adipocyte differentiation inhibitory activity and peroxisome proliferator activated receptor alpha (PPARα) activation effects. However, the bioactive constituents and functions of *Fraxinus mandshurica* seeds have not been studied. In the present study, we investigated the secoiridoid compounds in *F. mandshurica* seed extract (**FM**) using column chromatography, ^1^H-NMR, ^13^C-NMR and HPLC-DAD methods. The pancreatic lipase inhibitory activities of isolated compounds were evaluated in vitro. Additionally, the anti-obesity and gut microbiota modulation effect of **FM** on high-fat diet-induced obesity in C57BL/6 mice were also studied in vivo. The results showed that 19 secoiridoids were isolated from **FM** and identified. The total content of secoiridoids in **FM** reached 181.35 mg/g and the highest content was nuzhenide (88.21 mg/g). All these secoiridoid compounds exhibited good pancreatic lipase inhibitory activity with inhibition rate ranged from 33.77% to 70.25% at the concentration of 100 μM. After obese mice were administrated with **FM** at 400 mg/kg.bw for 8 weeks, body weight was decreased by 15.81%. Moreover, **FM** could attenuate the lipid accumulation in serum and liver, relieve the damage in liver and kidney, and extenuate oxidative stress injury and inflammation caused by obesity in mice. **FM** could also modulate the structural alteration of gut microbiota in obese mice, increasing the proportion of anti-obesity gut microbiota (*Bacteroidetes*, *Bacteroidia*, *S24-7* and *Allobaculum*), and reducing the proportion of obesogenic gut microbiota (*Firmicutes* and *Dorea*). This study suggests that *F. mandshurica* seeds or their secoiridoids may have potential for use as a dietary supplement for obesity management.

## 1. Introduction

With widespread social and lifestyle changes, obesity has become a serious public health issue. Obesity can cause many chronic diseases and metabolic disorders such as hyperlipidaemia [1], hypertension [2], cardiovascular diseases [3] type 2 diabetes [4] and certain cancers [5]. Currently, there are approximately 641 million obese individuals worldwide. China has the largest number of obese people and the prevalence of obesity in children and adolescents has been increasing [6]. By 2030, 49.5 million children will suffer from obesity, adversely affecting their physical and intellectual development [7,8]. Therefore, there is an urgent need to reduce both its prevalence and impact. Current strategies for obesity treatments including exercise, dietary control, life-style changes, prescription weight-loss medications and surgeries [9]. Among these options, appropriate diet and lifestyle changes plus moderate exercise are generally considered the most ideal ways to lose weight [10]. However, several clinical and epidemic studies have shown that maintaining long-term lifestyle modification is a great challenge to obese patients [11]. Pharmacotherapy can provide an easy way to accelerate weight loss, hence natural supplements derived from plants or synthetic chemicals have become a better choice for obesity treatment [12,13,14,15].

*Fraxinus mandshurica* Rupr., belonging to the Oleaceae family, is one of the most widely used hardwood trees in Northeast China, especially in the area of Changbai Mountain [16]. The dried bark of *F. mandshurica* has been used as traditional Chinese medicine for the treatment of inflammatory, fever, rheumatism arthritis and urinary retention [17]. Phytochemical investigations on *F. mandshurica* revealed that secoiridoids, phenylethanoids, hydroxycoumarins and lignans were its major constituents [18,19]. Among them, secoiridoids seem to be the most promising agents for obesity treatment. Ahn et al. have proved that four secoiridoid compounds from the stem barks of *Fraxinus rhynchophylla* exerted inhibitory effect on pancreatic lipase in a mixed mechanism of competitive and noncompetitive manners [20]. Nuzhenide and GI3, the primary secoiridoids in the extract from *Fraxinus excelsior* seed (FXE), could dose-dependently activate peroxisome proliferator-activated receptor alpha (PPARα) and inhibit preadipocyte differentiation in the 3T3-L1 cell model [21]. In addition, Ibarra et al. found that mice fed a high-fat diet containing 0.5% FXE had significantly lower body weight, omental and retroperitoneal fat levels at the end of the 16-week study compared to mice fed a high-fat diet alone [22]. The anti-obesity effect of FXE in obese Zucker rat model have also been studied. Results showed that chronic treatment with FXE at 100 mg/kg.bw per day for 5 weeks resulted in a significant decrease in glycemia (16.3%), triglyceridemia (33.4%) and body weight (8.1%) [23]. For the safety of FXE, in vitro, in vivo and clinical studies have clearly demonstrated that it is safe and well tolerated in healthy subjects [24].

*F. mandshurica* and *F. excelsior*, as two major species of *Fraxinus* genus, their seeds have similar chemical constituents [25]. Hence, we speculated that *F. mandshurica* seeds possessed beneficial effects against obesity. In this study, we investigated the secoiridoid compounds extracted from *F. mandshurica* seeds by MS, ^1^H-NMR, ^13^C-NMR and HPLC-DAD, and their pancreatic lipase inhibitory activity in vitro. In addition, the anti-obesity and gut microbiota modulation effects of secoiridoid-enriched extract obtained from the seeds of *F. mandshurica* on high-fat diet induced obese mice were studied. This study may provide references to the development of nutritional products of *F. mandshurica* seeds or their secoiridoids.

## 2. Results

### 2.1. Identification of Secoiridoids in F. mandshurica Seeds

**Figure 1:**
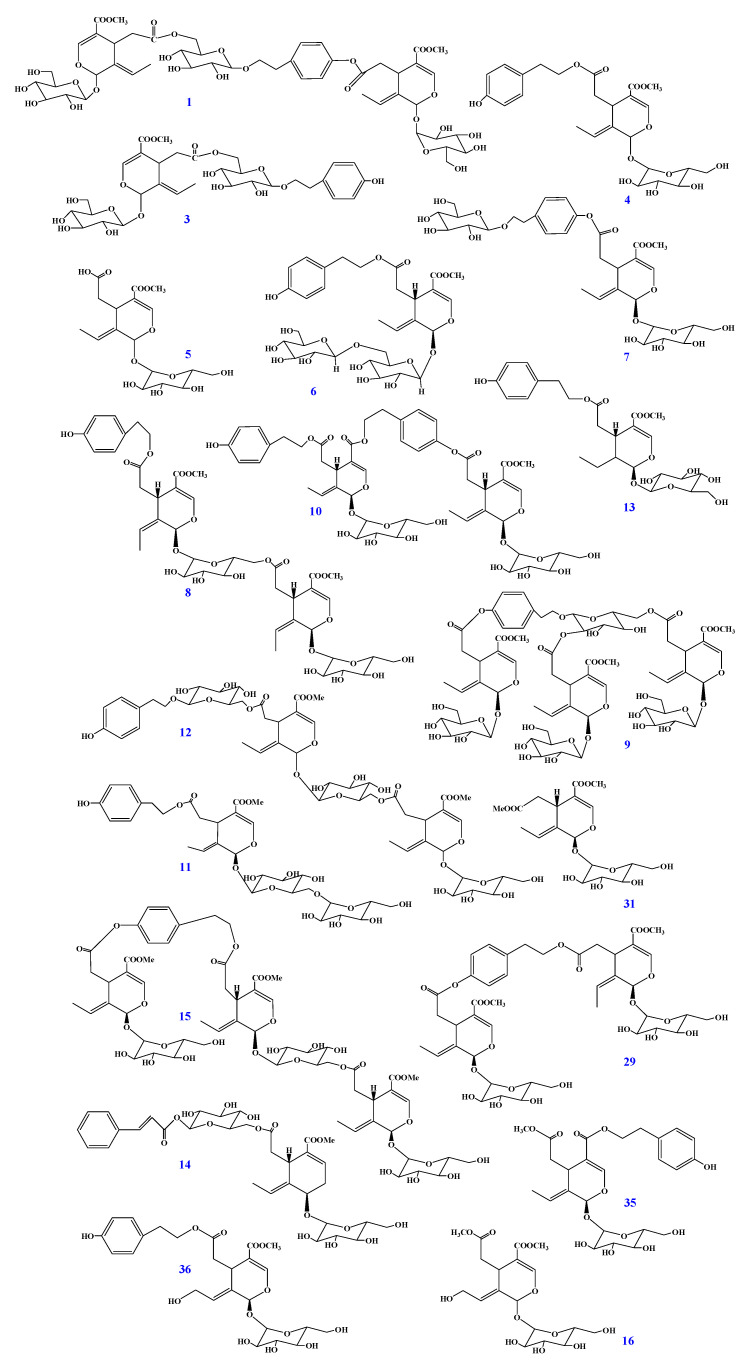
Chemical structures of secoiridoids in *F. mandshurica* seeds.

Nineteen secoiridoid compounds were first isolated from *F. mandshurica* seeds using a combination of column chromatography on silica gel, MCI GEL CHP-20P and polyamide. The structures of these secoiridoids were established by MS, ^1^H- and ^13^C-NMR and verified by comparing the NMR data (see the Supplementary Materials) with values reported in the literature. They were thus identified as GI3 (**1**) [26], nuzhenide (**3**) [27], ligstroside (**4**) [28], oleoside-11-methyl ester (**5**) [29], oleuricine A (**6**) [30], nicotiflorine (**7**) [31,32], jaspolyanoside (**8**) [33], oleopolynuzhenide A (**9**) [34], safghanoside G (**10**) [35], excelside B (**11**) [21], isooleonuezhenide (**12**) [36], lucidumoside A (**13**) [37], safghanoside A (**14**) [38], jaspolyoleoside B (**15**) [38], 10-hydroxoleoside-7,11-dimethyl ester (**16**) [39], GI5 (**29**) [40], oleoside-7,11-dimethyl ester (**31**) [41], isolignstroside (**35**) [42], 10-hydroxyligstroside (**36**) [43]. All the secoiridoids were found in *F. mandshurica* seeds in the form of glycosides. Their chemical structures are shown in Figure 1.

### 2.2. Quantitation of Secoiridoids in FM by HPLC Analysis

The quantitation results of nineteen secoiridoids in **FM** are shown in Table 1. All calibration curves exhibit excellent linear regressions with the determination coefficients (r^2^) ranging from 0.9986 to 1.0000. Total content of nineteen secoiridoids was 181.35 mg/g, the highest content was 88.21 mg/g (nuzhenide, **3**) and the lowest was 0.22 mg/g (isolignstroside, **35**).

### 2.3. Inhibitory Effect of Secoiridoids on Pancreatic Lipase

All secoiridoids were evaluated for their inhibitory effects on pancreatic lipase in vitro for the first time. The results are summarized in Table 1. Overall, the secoiridoids isolated from *F. mandshurica* seeds exhibited good pancreatic lipase inhibitory activity at the concentration of 100 μM. Oleopolynuzhenide A (**9**) showed the strongest inhibition among these secoiridoids, reaching 70.25%, and it was close to the positive control compound orlistat (73.11%). Further, by examining the structural characteristics of these secoiridoid compounds, we could conclude that compounds with two or three secoiridoid skeletons exhibit stronger inhibitory activity than compounds with only one basic skeleton. Specifically, inhibitory effects of GI3 (**1**), jaspolyanoside (**8**), oleopolynuzhenide A (**9**), safghanoside G (**10**), isooleonuezhenide (**12**), jaspolyoleoside B (**15**) and GI5 (**29**) were generally stronger than nuzhenide (**3**), ligstroside (**4**), oleoside-11-methyl ester (**5**), oleuricine A (**6**), nicotiflorine (**7**), excelside B (**11**), lucidumoside A (**13**), safghanoside A (**14**), 10-hydroxoleoside-7,11-dimethyl ester (**16**), oleoside-7,11-dimethyl ester (**31**), isolignstroside (**35**) and 10-hydroxyligstroside (**36**).

### 2.4. ***FM*** Declined the Body Weight of High-Fat Diet-Induced Obese Mice

**Figure 2:**
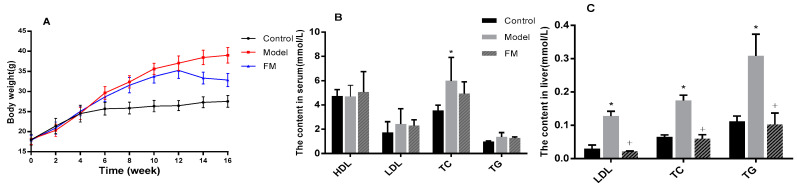
Effects of **FM** on body weight (**A**), TC, TG, LDL-C and HDL-C levels in serum (**B**) and TC, TG and LDL-C levels in liver (**C**). * *p* < 0.05 vs. control group. ^+^ *p* < 0.05 vs. model group.

Body weight was traced in this study and the results are displayed in Figure 2A. During the 16-week experiment period, all the mice were weighed every two weeks. At the end of the 8th week, the weight of the mice with high-fat diet (model group) reached 32.40 g, while the weight of the mice with low-fat diet (control group) were only 25.86 g. After administration with **FM** (400 mg/kg.bw) for the next 8 weeks, the body weight of the mice in FM group declined by 15.81% over the model group.

### 2.5. Effects of FM on TC, TG, LDL-C and HDL-C Levels in Serum and Liver

Results of TC, TG, LDL-C and HDL-C levels in serum are shown in Figure 2B. Compared to the control group, TC, TG and LDL-C levels of model group were increased by 68.4%, 39.4% and 39.7%, respectively. After treatment with **FM** at a dose of 400 mg/kg.bw for 8 weeks, the contents of TC, TG and LDL-C declined by 17.3%, 6.9% and 5.5% over the model group, respectively, and there was no significant difference (*p* > 0.05). Although high-fat diet caused a slight decrease of HDL-C content, **FM** consumption decreased HDL-C level compared to the model group (*p* > 0.05).

As shown in Figure 2C, the contents of TC, TG and LDL-C in the liver were significantly increased by high-fat diet feeding. A 167.5% increase of TC content, a 175.7% increase of TG content and a 331.8% increase of LDL-C content in liver were observed in the model group (*p* < 0.05). Notably, **FM** administration significantly decreased TC, TG and LDL-C levels by 65.9%, 66.8% and 83.0%, respectively, compared to the model group (*p* < 0.05).

### 2.6. Effects of FM on Liver Injury, Oxidative Stress and Inflammatory Factors

**Figure 3:**
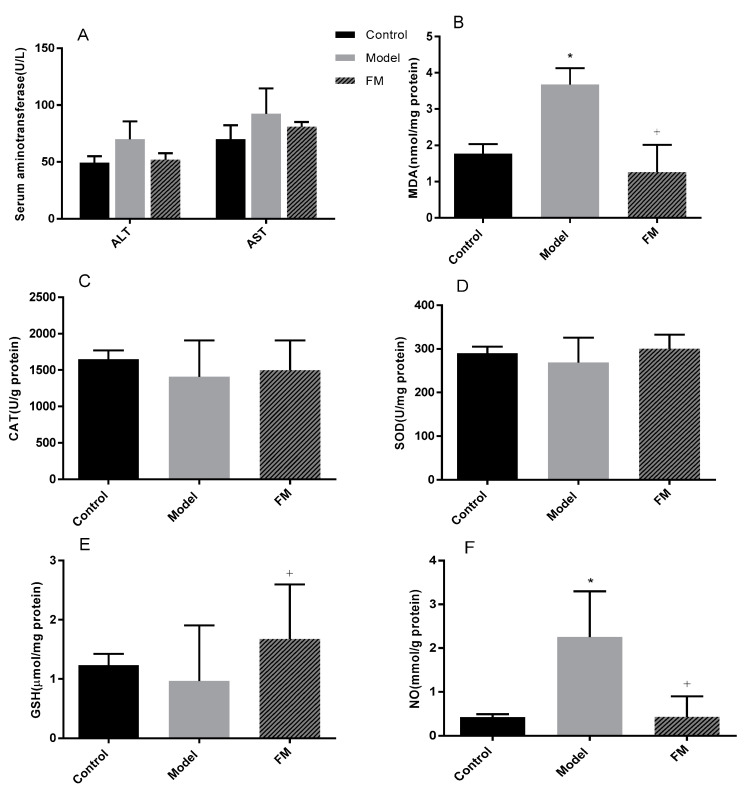
Effects of **FM** on serum ALT and AST activities (**A**), hepatic MDA content (**B**), CAT activity (**C**), SOD activity (**D**), GSH (**E**) and NO (**F**) contents. * *p* < 0.05 vs. control group. ^+^*p* < 0.05 vs. model group.

As shown in Figure 3A, ALT and AST levels were increased by feeding of a high-fat diet. ALT and AST levels in the model group were 69.97 and 92.56 U/L, 1.42 and 1.32 times that of the control group, respectively. ALT and AST in the levels FM group were mitigated after treatment with **FM**, and there was no statistically significant difference (*p* > 0.05).

In this study, hepatic oxidative stress was evaluated and the results are summarized in Figure 3B–F. A 7.4% decrease of SOD level and a 14.4% decrease of CAT level were observed in the model group compared with the control group. After treatment with **FM** for 8 weeks inhibited the high-fat diet-induced decrease in SOD and CAT levels in mouse liver was inhibited, but there was no statistically significant difference. MDA and NO contents were increased by feeding of a high-fat diet. MDA and NO contents in the model group were 2.08 and 5.23 times that of the control group, respectively (*p* < 0.05). However, administration with **FM** significantly reduced MDA and NO productions compared with the model group (*p* < 0.05). GSH content was decreased in model group fed with high-fat diet, showing about a 21.76% decline compared with the control group (*p* > 0.05). Compared with the model group, **FM** consumption increased the GSH content by 73.74% (*p* < 0.05).

**Figure 4:**
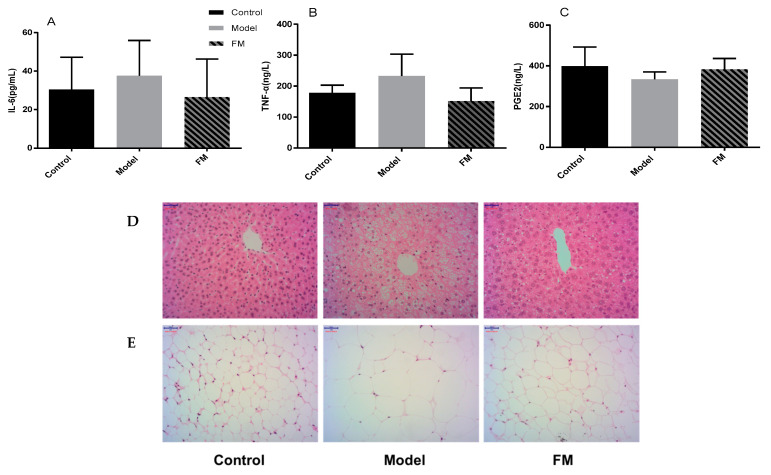
Effects of **FM** on serum IL-6 (**A**), TNF-α (**B**) and PGE2 (**C**) contents. * *p* < 0.05 vs. control group. ^+^ *p* < 0.05 vs. model group. Pathological sections of liver tissue (**D**) and epididymal adipose (**E**) in mice (H&E staining × 200).

Inflammatory factors including IL-6, TNF-α and PGE2 were also determined in this study, and the results are displayed in Figure 4A–C Compared to the control group, high-fat diet induced a 28.47% increase of IL-6 level, a 30.77% increase of TNF-α level and a 16.09% decrease of PGE2 level in the model group (*p* > 0.05). Meanwhile, 400 mg/kg.bw of **FM** consumption induced a 29.57% decrease of IL-6 level, a 34.67% decrease of TNF-α level and a 14.46% increase of PGE2 level compared with the model group (*p* > 0.05).

### 2.7. Histopathological Examinations of Liver and Epididymal Adipose

Morphological analysis of liver and epididymal adipose were conducted. As shown in Figure 4D, mice in the control group fed with low-fat diet showed normal hepatic tissue with regular morphology of hepatic cells, while, mice in the model group fed with high-fat diet exhibited macrovesicular steatosis and cell ballooning degeneration. After administration with **FM**, pathological changes of mice in the FM group were effectively reversed. The results of morphological analysis of epididymal adipose are shown in Figure 4E, the adipocytes in the model group were much bigger than the control group, administration of **FM** alleviated the adverse change.

### 2.8. Effects of FM on Serum Cr, BUN and UA Levels

**Figure 5:**
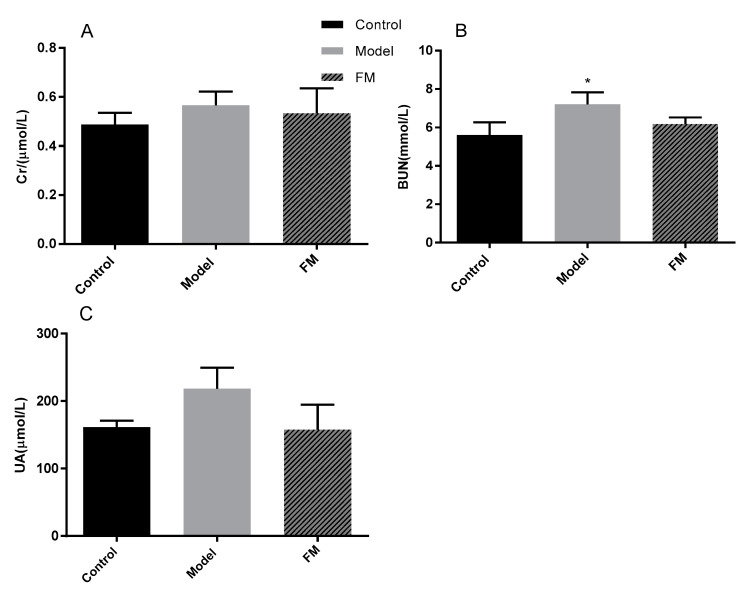
Effects of **FM** on serum Cr (**A**), BUN (**B**) and UA (**C**) levels. * *p* < 0.05 vs. control group.

Cr, BUN and UA levels were determined to evaluate the kidney status of mice. As shown in Figure 5A–C, mice in the model group showed a significant increase of BUN level compared with the mice in the control group (*p* < 0.05). And high-fat diet induced a 16.05% increase of Cr level and a 35.41% increase of UA level in the model group compared with the control group, and there was no statistically significant difference (*p* > 0.05). After administration with **FM**, the contents of Cr, BUN and UA in the FM group were decreased, and there was no statistically significant difference (*p* > 0.05).

### 2.9. FM Modulates the Gut Microbiota in High-Fat Diet-Induced Obese Mice

**Figure 6:**
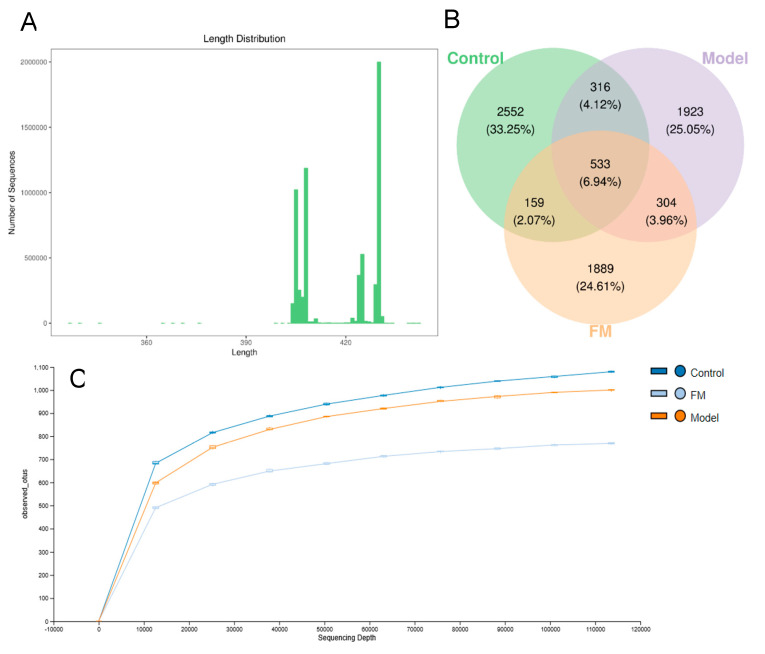
Sequence number and length of samples (**A**), Venn diagram for describing the common and unique OTUs (**B**) and rarefaction curve of OTUs of samples (**C**).

To evaluate the impact of **FM** on the gut microbiota in mice, pyrosequencing of the variable region V3-V4 of the bacterial 16S rRNA gene was performed in this study. As shown in Figure 6A, a total of 3,682,083 sequences (400–450 base pairs in length) were obtained from 22 samples, including an average of 162,037 sequences in the control group, 172,220 sequences in the model group and 168,002 sequences in the FM group. The Venn diagram (Figure 6B) shows that the qualified sequences (>0.001%) were clustered into 7676 bacterial OTUs. Among them, 316 same OTUs were identified in the control and model groups, 159 same OTUs were found in the control and FM groups. Administration with **FM** decreased the shared OTUs in the mice of the control and model groups. From Figure 6C, we can conclude that rarefaction curves of three groups tend to be flat when the sequencing depth increases. This indicated that the sequencing results were sufficient to reflect the gut microbial diversity of the samples.

**Figure 7:**
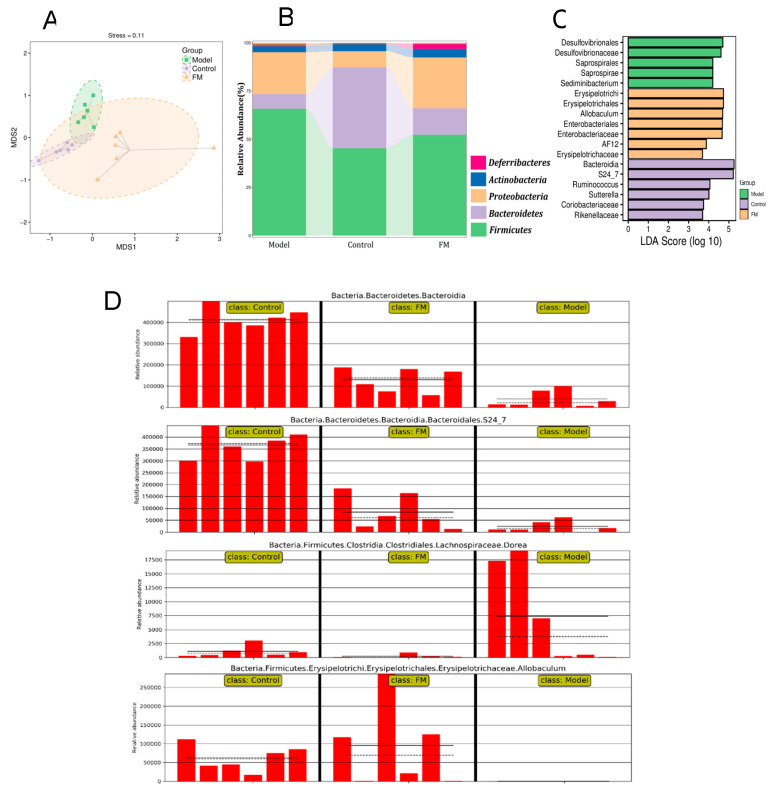
The NMDS analysis (**A**), the relative abundance (%) of bacterial at phylum level in different groups (**B**), the significantly different species in three groups (**C**) and the relative abundance the of gut microbial biomarkers in three groups (**D**).

Non-metric multidimensional scaling (NMDS) analysis based on UniFrac distance was performed to evaluate the similarity of gut microbial communities between different groups. As shown in Figure 7A, the similarity was described by the distance matrix of different samples. Samples in different groups clustered separately, the sample distributions of the model group and FM group were far away from the control group. This indicated that consumption of a high-fat diet and **FM** changed the gut microbiota composition. And samples in the FM group exhibited a unique gut microbial structure. The stress value is 0.11 (<0.2), indicating that the analysis results were reliable. The relative abundance of major microbiota constituents at phylum level is shown in Figure 7B, *Firmicutes*, *Bacteroidetes* and *Proteobacteria* were the top three phyla. Compared to the control group, the high-fat diet caused an increase of *Firmicutes* and a decrease of *Bacteroidetes* in the model group, while, **FM** consumption alleviated these changes in the FM group. The impact of **FM** on the key community of gut microbiota was described by histogram of LDA value (Figure 7C). *Bacteroidia*, *S24-7*, *Ruminococcus* and *Sutterella* were the four main communities in the gut of the control mice. The high-fat diet induced a significant effect on *Desulfovibrionales*, *Desulfovibrionaceae*, *Saprospirales*, and *Saprospirae* in the gut of the model mice. However, **FM** consumption demonstrated a selective enrichment of *Erysipelotrichi*, *Erysipelotrichales*, *Allobaculum*, *Enterobacteriales* and *Enterobacteriaceae.* The relative abundance of gut microbial biomarkers of three groups are displayed in Figure 7D. To visually show the differences between three groups, the solid and dashed lines are used to identify the average and median relative abundance of the taxa in each group. In the control group, the abundance of *Bacteroidia* and *S24-7* were high, however, high-fat diet induced significant increase of those in the model group. Adversely, the abundance of *Dorea* was low in the control group, and it was greatly increased by high-fat diet in the model group. Notably, compared with the control group, the high-fat diet completely inhibited the growth of *Allobaculum*. Treatment with **FM** could significantly alleviate the changes as mentioned above.

## 3. Discussion

Obesity and overweight are major contributors to the global burden of chronic diseases and complications [44]. Thus, edible and medicinal plants and their bioactive components helping people to fight the battle against obesity have been widely explored, such as the extracts of garcinia, green tea and white bean [13,14,15]. The present investigation showed that *F. mandshurica* seed extract containing secoiridoids reduced body weight and lipid levels in serum and liver, alleviated liver and kidney damage and regulated the gut microbiota in high-fat diet-induced obese mice.

Studies in recent years have demonstrated that secoiridoid-enriched extract from *F. excelsior* seed reduced gains in body weight, omental fat and retroperitoneal fat in obese C57BL/6J mice, and alleviated triglyceridemia, body weight gain and systolic blood pressure in SHR and Zucker rats [22,23]. In the present study, treatment with **FM** for 8 weeks reduced body weight in obese mice. Serum and liver TG, TC, LDL-C contents in high-fat diet-induced obese mice were increased and **FM** consumption reduced these lipid levels. Obesity can cause a decrease in HDL-C concentration and HDL-C dysfunction, in this study, the serum HDL-C content of mice treated with **FM** was higher than that of obese mice.

Obesity is usually associated with chronic metabolic diseases such as nonalcoholic fatty liver disease [45]. Ibarra et al. have proved that extract from *F. excelsior* seed lowered liver weight gains and the incidence of fatty livers in obese C57BL/6J mice [22]. ALT and AST levels are clinically considered as credible markers for monitoring liver injury or liver function [46,47]. The present investigation showed that high-fat diet induced increases of serum ALT and AST levels in obese mice, and after treatment with **FM** for 8 weeks, the above variations were alleviated.

Obesity usually accompanied by increased oxidative stress caused by the reactive oxygen species (ROS). High-fat diet can also produce ROS by changing oxygen metabolism, which further aggravates the oxidative stress in an obese body [48]. Finally, persistent oxidative stress leads to the consumption of antioxidants and the decreased levels of antioxidant enzymes such as CAT and SOD [49]. Younis et al. reported that *F. xanthoxyloides* leaf extract increased the level of hepatic antioxidant enzymes including CAT, SOD, peroxidase (POD), glutathione-*S*-transferase (GST) and glutathione reductase (GSR) in CCl_4_- induced hepatotoxic rats [50]. In the present study, administration of **FM** significantly increased the levels of CAT and SOD in high-fat diet-induced obese mice. Obesity-related mitochondrial dysfunction can cause increased NO synthesis [51]. After treatment with **FM** for 8 weeks, the NO content of high-fat diet-induced obese mice was significantly decreased and was close to that of the low-fat diet mice. Additionally, GSH content of high-fat diet induced obese mice was significantly increased after administration with **FM**. GSH can function as direct free-radical scavenger and reduce intracellular ROS, thereby protect the cell against toxicity and disease [52]. MDA is the main product of lipid peroxidation induced by ROS, its content in high-fat diet-induced obese mice was significantly decreased by the treatment of **FM** [53]. Inflammation is a manifestation of increased oxidative stress induced by obesity [48]. Recent study has indicated that ROS generated during fatty acid oxidation triggers inflammatory pathway involving TNF-α and IL-6 [54]. Younis et al. also proved that *F*. *xanthoxyloides* leaf extract had the ability to decrease the concentrations of TNF-α and IL-6 in air pouch exudate of rat [55]. In the present study, TNF-α and IL-6 levels were increased in high-fat diet induced obese mice, and administration of **FM** to obese mice for 8 weeks significantly decreased the TNF-α and IL-6 contents. PGE2, an anti-inflammatory lipid mediator derived enzymatically from arachidonic acid and catalyzed by cyclooxygenase (COX-1 and -2) and prostaglandin E synthase, which inhibits the differentiation and activation of inflammatory cells [56]. A decreased level of PGE2 have been observed in high-fat diet-induced obese mice, after treatment with **FM** for 8 weeks, the PGE2 content was increased. Overall, the above results illustrated that *F. mandshurica* seed extract significantly inhibited the obesity and alleviated liver injury, oxidative stress and inflammation induced by high-fat diet. The beneficial effects of *F. mandshurica* seed on obesity and obesity-related liver injury were also confirmed by conventional histological assessment of adipocytes and livers.

A report has demonstrated that obesity is associated with increased risk of developing chronic kidney disease [57]. The present study demonstrated that **FM** alleviated kidney injury as indicated by the decreased levels of serum Cr, BUN and UA in high-fat diet induced obese mice.

In recent years, the impact of gut microbiota on obesity has become a hot research topic. Generally, the gut microbiota consists of six bacterial phyla, including *Firmicutes*, *Bacteroidetes*, *Protebacteria*, *Actinobacteria*, *Fusobacteria* and *Verrucomicrobia*. Among them, *Firmicutes* and *Bacteroidetes* account for 90% and are closely related to obesity [58,59]. Hildebrandt et al. reported that a decrease in *Bacteroidetes* and an increase in *Firmicutes* were found in the high-fat diet induced obese mice, we also observed these alterations in obese mice in this study [60]. Interestingly, **FM** consumption can increase the abundance of *Bacteroidetes* and decrease the abundance of *Firmicutes*, the result preliminarily shows that **FM** can treat obesity by regulating the gut microbiota. Further, we found several gut microbial biomarkers between three groups, including *Bacteroidia*, *S24-7*, *Dorea* and *Allobaculum.* In this study, the decrease in relative abundance of *Bacteroidia* in high-fat diet mice agrees with a previous study [61]. After treatment with **FM** for 8 weeks, *Bacteroidia* were enriched and were negatively correlated with obesity phenotypes. *S24-7*, a bacterium that produces butyrate, has been shown to be beneficial to intestinal epithelial health and energy metabolism [62,63]. However, compared to the low-fat diet mice, the abundance of *S24-7* in high-fat diet obese mice was significantly decreased, and **FM** consumption promoted an increase of *S24-7* in obese mice. Studies have found that *Dorea* is positively correlated with body mass index (BMI), waist circumference and diastolic blood pressure. Additionally, a high abundance of *Dorea* was found in the intestines of patients with multiple sclerosis and inflammatory bowel disease, suggesting that a pro-inflammatory effect of this genus [64,65]. Compared with the low-fat diet mice, the abundance of *Dorea* in high-fat diet induced obese mice was significantly increased, and it could be obviously decreased by consumption of **FM**. It has been reported that *Allobaculum* was negatively associated with obesity, inflammation and insulin resistance [66]. Notably, in the present study, the *Allobaculum* of high-fat diet induced obese mice was completely inhibited, and the **FM** consumption effectively increases its abundance. The above results indicate that the modulation of gut microbiota caused by **FM** consumption has a therapeutic effect on obesity in mice.

To identify the bioactive constitutes of **FM**, extensive chromatographic and spectral analysis were used in the phytochemical study. Finally, we obtained nineteen secoiridoid compounds from the **FM** for the first time. The content of each secoiridoid in **FM** is quantified by a RP HPLC-DAD method and quantitative results ranged from 0.22 to 88.21 mg/g. Nuzhenide was the richest among these secoiridoids, which has been shown to activate PPARα and inhibit preadipocyte differentiation in the 3T3-L1 cell [21]. In the human body, fat is hydrolyzed by pancreatic lipase and then absorbed in the small intestine, therefore, pancreatic lipase inhibitors can be used to prevent/treat obesity [67]. Interestingly, nineteen secoiridoids isolated from **FM** exerted good pancreatic lipase inhibitory activity in vitro. Hence, the anti-obesity effect of **FM** should be attributed to the abundant secoiridoid compound. However, the mechanism of the anti-obesity effect of secoiridoids in **FM** was not clear and this will require more studies to be performed.

## 4. Materials and Methods

### 4.1. Preparation of F. Mandshurica Seed Extract

The dried seeds of *F. mandshurica* were purchased from Jilin City, Jilin Province, China in 17 November 2017. The botanical origins were identified by the corresponding author Prof. Naisheng Bai, and voucher specimens of *F. mandshurica* seeds (FM20171117) were deposited in Room 611, College of Food Science and Technology, Northwest University. The plant materials (30.0 kg) were cleaned and powdered (60–100 mesh), then percolated three times (24 h each time) with 75% ethanol-water solution at room temperature, the ratio of the material to solvent was 1:3 (*w*/*v*). The ethanol solution was directly concentrated under vacuum at less than 50 °C. Then the residue was dispersed in water and partitioned between methylene chloride three times. After removing solvent, the water portion was dried under vacuum below 50 °C to obtain powder extract (**FM**). Sample **FM** was subjected to the isolation and characterization of secoiridoids, HPLC analysis and animal study.

### 4.2. Extraction and Isolation of Secoiridoid Compounds from F. mandshurica Seeds

The **FM** (540 g) residue was chromatographically separated on a polyamide column (PA, 60–100 mesh, 2000 g) and eluted with an EtOH/H_2_O gradient solvent system. The effluent was combined after monitoring by TLC and seven major fractions (I–VII) were collected.

Sample I (94.3 g) was chromatographed on a PA column (60–100 mesh, 300 g) and the eluent was EtOH/H_2_O gradient solvent system, then three samples of different polarity were collected (I-1, I-2 and I-3). Compounds **9** (5.4 mg), **12** (25.0 mg) and **13** (13.2 mg) were isolated from I-1 and I-2 using a CHP20P column (80 g) and the eluent was MeOH/H_2_O system. I-3 was successively chromatographed on a CHP20P column (50 g, MeOH/H_2_O, 1:1), a silica gel column (200–300 mesh, 300 g, CH_2_Cl_2_/MeOH, 20:1–1:1) and a CHP20P column (50 g, MeOH/H_2_O, 1:1), to give pure compounds **11** (8.1 mg) and **7** (15.9 mg).

Sample II (44.1 g) was submitted to a silica gel column (200–300 mesh, 500 g, CH_2_Cl_2_/MeOH, 40:1–1:1) and three sub fractions were collected. Compound **15** (11.3 mg) was purified by a CHP20P column (100 g, MeOH/H_2_O, 0:1–1:0) from II-1. Isolation of compound **10** (9.5 mg) from II-2 was effected by a silica gel column (200–300 mesh, 500 g, CH_2_Cl_2_/MeOH, 20:1–1:1) and a CHP20P column (100 g, MeOH/H_2_O, 1:1). II-3 was repeatedly chromatographed on silica gel columns (300–400 mesh, 500 g, 20:1–1:1) to give pure compound **6** (28.3 mg)

Sample III (22.8 g) was chromatographed on a CHP20P column (500 g, MeOH/H_2_O, 0:1–1:0) to give two polar parts. Further, one part (III-1) was chromatographed on a silica gel column (300–400 mesh, 300 g, CH_2_Cl_2_/MeOH, 20:1–1:1) to afford pure compound **8** (18.1 mg); the other part (III-2) was chromatographed on a CHP20P column (100 g, MeOH/H_2_O, 1:1) to yield pure compound **14** (6.4 mg).

Sample IV (35.7 g) was successively separated by a PA column (100–200 mesh, 500 g, EtOH/H_2_O, 0:1–1:0), a silica gel column (200–300 mesh, 500 g, CH_2_Cl_2_/MeOH, 20:1–1:1) and a CHP20P column (300 g, MeOH-H_2_O, 0:1–1:0) to give pure compounds **4** (8.9 mg), **1** (11.3 mg) and **3** (16.7 mg).

Sample V (62.4 g) was repeatedly chromatographed on CHP20P columns (500 g, MeOH/H_2_O, 0:1–1:0) to afford pure compounds **16** (10.1 mg), **35** (6.7 mg) and **29** (14.1 mg).

Sample VI (78.6 g) was repeatedly chromatographed on silica gel column (200–300 mesh, 800 g, CH_2_Cl_2_/MeOH, 30:1–1:1) to give VI-1 and VI-2. Compound **5** (21.3 mg) was purified from VI-1 using a CHP20P column (500 g, MeOH/H_2_O, 0:1–1:0). The isolation of compound **36** (15.5 mg) from VI-2 was orderly effected by a silica gel column (200–300 mesh, 100 g, CH_2_Cl_2_/MeOH, 20:1–1:1) and a CHP20P column (100 g, MeOH/H_2_O, 2:1).

Sample VII (67.7 g) was submitted to repeated silica gel columns (300–400 mesh, 500 g, CH_2_Cl_2_/MeOH, 25:1–1:1) for the separation of compound **31** (17.3 mg).

The separation process of pure compounds is shown in Figure S1 (Supplementary Materials). These compounds were identified by MS, ^1^H- and ^13^C-NMR.

### 4.3. Quantification of Secoiridoids in ***FM*** by HPLC Analysis

HPLC analysis was performed on an Agilent 1260 LC Series instrument (Agilent Technologies, Santa Clara, CA, USA) connected to a G4212B DAD detector, using an Agilent Eclipse Plus C_18_ column (250 × 4.6 mm, 5 μm) with a flow rate of 1.0 mL/min, and column temperature was maintained at 30 °C. The mobile phase was composed of D (water) and C (acetonitrile) with a gradient elution: 0 min, 95% D; 0–8 min, 95–75% D; 8–18 min, 75–50% D; 18–28 min, 50–25% D; 28–40 min, 25–15% D; 40–52 min, 15–5% D; 52–58 min, 5% D; 58–60 min, 5–95% D; 60–70 min, 95% D. The chromatogram was monitored at a wavelength of 220 nm during the experiment. Nineteen chemical standards used in quantitative analysis were isolated from **FM** and purity of each standard compound was over 98% as monitored by HPLC analysis. The standard solution containing nineteen secoiridoids were prepared and diluted to five different concentrations. Calibration curve for each secoiridoid was constructed by plotting peak area *versus* corresponding concentration. The sample solution was obtained by dissolving **FM** in MeOH. Secoiridoids were identified by comparing their UV spectra and HPLC retention times of target peaks with those of standard compounds.

### 4.4. Pancreatic Lipase Inhibitory Activity of Secoiridoids In Vitro

Pancreatic lipase inhibitory activities of secoiridoids were determined as previously reported [68,69]. Briefly, the enzyme solution was prepared by the reconstitution of porcine pancreatic lipase (Sigma-Aldrich, St. Louis, MO, USA) in 0.1 M Tris-HCl buffer (pH = 8). Then, all isolated compounds (100 μM) and reference compound orlistat (100 μM) and different concentrations of **FM** were mixed with enzyme buffer and incubated for 20 min at 37 °C. After incubation, 10 mM *p*-nitrophenyl butyrate (*p*-NPB) was added at 37 °C to start the reaction. The absorbance at 405 nm was determined for 20 min with interval of 2 min using a BioTek Epoch microplate spectrophotometer (Winooski, VT, USA). The enzyme activity was represented by the absorbance growth slope (K) which was calculated by linear regression. The inhibitory rate was calculated by the formula below:

% Inhibition = (K_b_ − K_s_)/K_b_ × 100 (1)

where K_s_ and K_b_ were the enzyme activity in the presence and absence of secoiridoids, respectively. Orlistat (Sigma-Aldrich) was used as a positive control. The IC_50_ values were determined by nonlinear regression with normalized dose-response fitting using Graphpad Prism 6 software (San Diego, CA, USA), which represent the concentrations of compounds at which 50% of the pancreatic lipase activity was inhibited.

### 4.5. Animal Experiments

#### 4.5.1. Animals and Diets

Thirty male C57BL/6 mice (20 ± 2 g) were purchased from the Experimental Animal Center of Xi’an Jiaotong University. All the mice were maintained at temperature of 25 ± 2 °C, relative humidity of 50 ± 10% and subjected to a 12 h light/dark cycle for 7 days of adaptation. Then thirty mice were randomly assigned to 3 groups with 10 mice per group. All animal experimental protocols used in this study were approved by the Regulations of Experimental Animal Administration issued by the State Committee of Science and Technology of People’s Republic of China (SCXK 2012-003). Mice in control group were fed with a low-fat diet (LFD, 8% fat, 22% proteins and 70% carbohydrates). Mice in other two groups were given a high-fat diet (HFD, 45% fat, 18% proteins and 37% carbohydrates) [70]. All mice subsisted on the experimental diets until the end of the experiment. After 8 weeks of daily feeding, one of the high-fat diet group (**FM** group) was given 400 mg/kg.bw **FM** by intragastric administration, another high-fat diet group was named as model group. After 16 weeks of treatment, all the mice were food-deprived for 10 h and decapitated. Whole blood was obtained by cardiac puncture. Liver, colon and epididymal adipose were separated and harvested. All samples were stored at −80 °C.

#### 4.5.2. Serum and Hepatic Biochemical Analysis

The levels of high density lipoprotein cholesterol (HDL-C), low density lipoprotein cholesterol (LDL-C), total cholesterol (TC), triglyceride (TG), alanine aminotransferase (ALT), aspartate aminotransferase (AST), creatinine (Cr), blood urea nitrogen (BUN) and uric acid (UA) in serum samples were measured with commercial diagnostic kits (Nanjing Jiancheng Bioengineering Institute, Nanjing, China).

Livers were immediately excised from sacrificed mice and washed with ice-cold physiological saline three times to remove the blood, fat and connective tissue. Then the liver from dried filter paper was weighed and homogenized with ice-cold physiological saline using a Dounce tissue grinder (Kimble, Vineland, NJ, USA) with 20,000 rpm for 10 s. Finally, the homogenate was centrifuged at 3000 rpm for 20 min at 4 °C and the supernatant was used for hepatic biochemical analysis. Malondialdehyde (MDA), catalase (CAT), superoxide dismutase (SOD), glutathione (GSH) and nitric oxide (NO) levels were determined using diagnostic kits (Nanjing Jiancheng Bioengineering Institute, Nanjing, China). The levels of tumor necrosis factor (TNF-α), prostaglandin E2 (PGE2) and interleukin-6 (IL-6) were detected using commercial ELISA kits (Shanghai Fusheng, Shanghai, China). In addition, the supernatant sample was used for determination of TG and TC contents. A multifunctional plate reader (Infinite M200Pro, TECAN, Männedorf, Switzerland) was used for the quantitative measurements.

#### 4.5.3. Histopathological Examinations of Liver and Epididymal Adipose

Liver and epididymal adipose tissues was immersed in 10% neutral formalin solution and embedded in paraffin. Then processed by standard procedures, paraffin-embedded section at 5 μm thickness was cut and stained with hematoxylin and eosin (H&E).

### 4.6. Gut Microbiota Analysis

Six samples of colonic contents per group were randomly selected for Illumina sequencing and statistical analysis of the bacterial 16S rRNA gene. Total DNA was extracted using the Fast SPIN extraction kits (MP Biomedicals, Santa Ana, CA, USA). Quantification of DNA was measured by a NanoDrop ND-1000 spectrophotometer (Thermo Fisher Scientific, Waltham, MA, USA) and the quality of DNA was evaluated by 1.2% agarose gel electrophoresis. The V3-V4 hypervariable regions of 16S rRNA genes were amplified using primers 338-forward (5′-ACTCCTACGGGAGGCAGCA-3′) and 806-reverse (5′-GGACTACHVGGGTWTCTAAT-3′). Quantification of the purified PCR amplicons was checked by PicoGreendsDNA Assay Kit (Invitrogen, Carlsbad, CA, USA). Then the purified PCR products were pooled in equimolar and paired-end sequenced (2 × 300 bp) on an Illlumina MiSeq platform with MiSeq Reagent Kit V3 (600 cycles) (Shanghai Personal Biotechnology Co., Ltd., Shanghai, China). The OTU (operational taxonomic unit) relative abundances were hierarchically clustered and visualized by the R software (v3.5.1) gplots package, and a LEfSe (linear discriminant analysis effect size) analysis coupled with the Kruskal-Wallis and Wilcoxon rank sum tests was performed.

### 4.7. Statistical Analysis

All experiments were performed in triplicate and results were represented as mean ± SD. Differences between control and treated groups were assessed by Duncan’s multiple range test after SAS one-way ANOVA, version 8.1 (SAS Institute, Cary, NC, USA). *p* < 0.05 was significantly different.

## 5. Conclusions

In conclusion, **FM** could reduce the body weight of obese mice, attenuate the lipid accumulation in serum and liver, relieve the damage in liver and kidney, and extenuate oxidative stress injury and inflammation caused by obesity. In addition, **FM** could modulate the structural alteration of gut microbiota in obesity mice, increase the proportion of anti-obesity gut microbiota (*Bacteroidetes*, *Bacteroidia*, *S24-7* and *Allobaculum*), and reduce the proportion of obesogenic gut microbiota (*Firmicutes* and *Dorea*). The anti-obesity and gut microbiota modulation effect could be associated with the abundant secoiridoid compounds in **FM**. The phytochemical study of **FM** resulted in the isolation of nineteen secoiridoid compounds. And these compounds exhibited good pancreatic lipase inhibitory activity in vitro. In summary, this study suggests that *F. mandshurica* seed or its secoiridoids may have potential for use as a dietary supplement for controlling obesity.

## Figures and Tables

**Table 1:** Contents and pancreatic lipase inhibitory activity of secoiridoids.

No.	Name	Calibration Curves ^a^	R^2^	Content ^b^ (mg/g)	Inhibition% ^b^ (100 μM)	IC50 ^b^ (μM for Compounds) (mg/L for FM Extract)
**1**	GI3	Y = 481.08 + 6.30X	0.9984	9.17 ± 0.05	53.42 ± 6.33	95.36 ± 3.12
**3**	Nuzhenide	Y = 667.40 + 4.41X	0.9999	88.21 ± 0.19	43.45 ± 3.28	120.97 ± 4.94
**4**	Ligstroside	Y = 40.34 + 13.69X	0.9986	16.88 ± 0.11	49.67 ± 4.25	100.59 ± 2.74
**5**	Oleoside-11-methyl ester	Y = 153.23 + 3.97X	0.9991	2.90 ± 0.03	48.53 ± 5.13	102.94 ± 4.13
**6**	Oleuricine A	Y = 32.99 + 2.35X	0.9997	0.42 ± 0.01	41.41 ± 1.95	126.27 ± 0.87
**7**	Nicotiflorine	Y = −65.95 + 4.79X	0.9999	7.37 ± 0.10	37.35 ± 6.34	135.84 ± 4.98
**8**	Jaspolyanoside	Y = 108.89 + 0.36X	0.9995	3.19 ± 0.02	62.41 ± 3.98	66.46 ± 2.20
**9**	Oleopolynuzhenide A	Y = 42.78 + 2.25X	1.0000	0.62 ± 0.03	70.25 ± 4.56	54.60 ± 2.38
**10**	Safghanoside G	Y = 91.52 + 2.39X	1.0000	5.55 ± 0.09	60.16 ± 5.78	67.91 ± 3.49
**11**	Excelside B	Y = 169.19 + 2.31X	0.9999	3.22 ± 0.02	49.98 ± 4.33	100.06 ± 2.26
**12**	Isooleonuezhenide	Y = 81.21 + 3.72X	0.9999	0.89 ± 0.12	57.62 ± 4.83	90.48 ± 3.67
**13**	Lucidumoside A	Y = 261.83 + 3.66X	0.9997	6.11 ± 0.01	47.69 ± 8.36	102.37 ± 5.02
**14**	Safghanoside A	Y = 130.90 + 1.87X	0.9996	4.81 ± 0.10	33.77 ± 6.14	193.81 ± 4.60
**15**	Jaspolyoleoside B	Y = −1.75 + 0.09X	0.9999	1.33 ± 0.05	53.32 ± 7.31	96.27 ± 4.91
**16**	10-hydroxoleoside-7,11-dimethyl ester	Y = −1578.72 + 15.65X	0.9993	5.31 ± 0.01	43.57 ± 3.09	117.42 ± 1.38
**29**	GI5	Y = −2.04 + 1.72X	0.9999	8.91 ± 0.01	58.77 ± 6.01	90.51 ± 2.24
**31**	Oleoside-7,11-dimethyl ester	Y = −26.33 + 15.08X	0.9998	10.79 ± 0.02	50.11 ± 5.97	99.78 ± 3.88
**35**	Isolignstroside	Y = 235.57 + 4.07X	0.9999	0.22 ± 0.03	52.07 ± 2.99	98.87 ± 2.85
**36**	10-hydroxyligstroside	Y = 6236.70 + 1.94X	0.9991	5.45 ± 0.01	36.35 ± 5.27	167.72 ± 3.51
**Positive**	Orlistat	/	/	/	73.11 ± 3.23	48.92 ± 2.44
**Extract**	FM	/		/	/	80.29 ± 1.28

^a^ Y is the value of peak area, X is the value of the compound’s concentration (μg/mL). ^b^ Data are represented as the mean ± SD (*n* = 3).

## Supplementary Materials

The following are available online, Figure S1: The separation process of *F. mandshurica* seeds, MS, 1H NMR and 13C NMR data of compounds.
