# Supplementary material for: Anti-Obesity and Gut Microbiota Modulation Effect of Secoiridoid-Enriched Extract from Fraxinus mandshurica Seeds on High-Fat Diet-Fed Mice
Source: Molecules. 2020 Sep 2;25(17):4001. doi: 10.3390/molecules25174001 (PMC7504722; doi:10.3390/molecules25174001)
Supplement: Supplementary file 1 [file molecules-25-04001-s001.pdf]

Supplementary Materials

Figure:

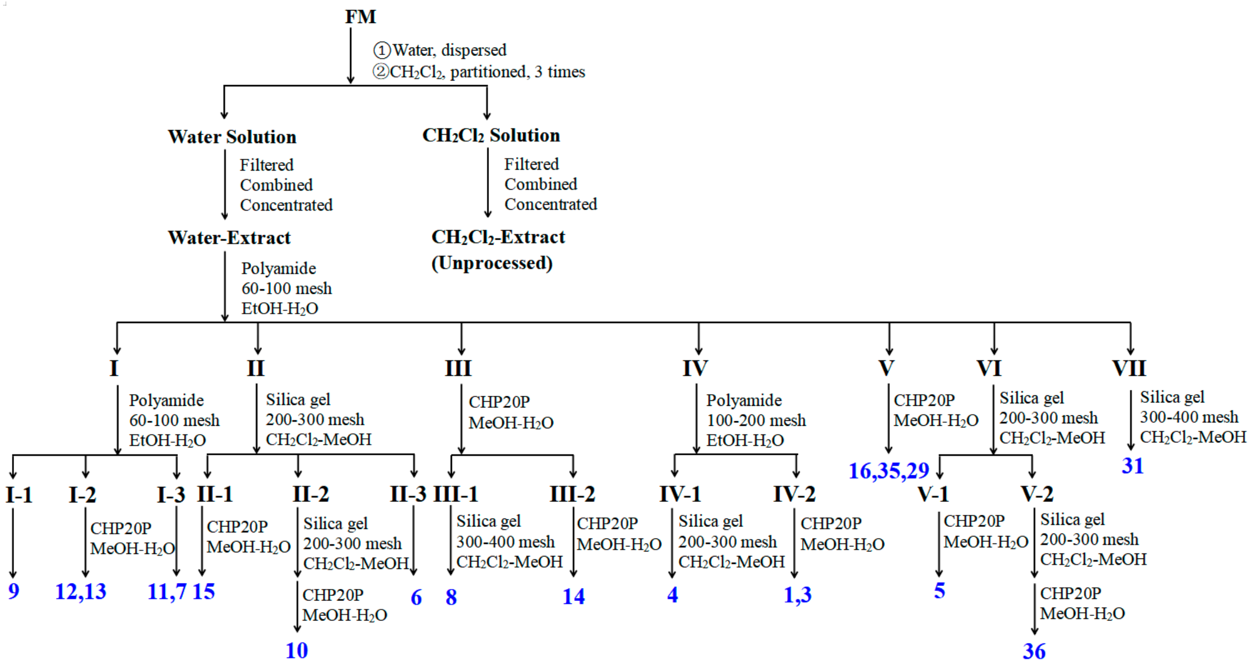

Fig. S1 The separation process of *F. mandshurica* seeds

NMR data:

GI3 (1): FAB-MS, m/z: 1060.02 $[M+H]^+$. ^{13}C NMR (400 MHz, CD_3OD-d_4): Part A δ_C 95.3 (1-C), 155.3 (3-C), 109.4 (4-C), 31.8 (5-C), 41.2 (6-C), 173.1 (7-C), 130.6 (8-C), 125.2 (9-C), 13.9 (10-C), 168.5 (11-C), 52.1 (11-OMe), 101.0 (1′-C), 75.0 (2′-C), 78.4 (3′-C), 71.5 (4′-C), 77.9 (5′-C), 62.7 (6′-C), 104.5 (1″-C), 74.7 (2″-C), 78.4 (3″-C), 71.6 (4″-C), 75.1 (5″-C), 65.7 (6″-C), 71.7 (1‴-C), 36.6 (2‴-C), 138.0 (3‴-C), 131.1 (4‴-C), 122.1 (5‴-C), 150.5 (6‴-C), 122.6 (7‴-C), 131.1 (8‴-C); Part B δ_C 95.2 (1-C), 155.3 (3-C), 109.3 (4-C), 31.8 (5-C), 41.1 (6-C), 171.8 (7-C), 130.5 (8-C), 125.0 (9-C), 13.8 (10-C), 168.5 (11-C), 52.1 (11-OMe), 100.8 (1′-C), 74.7 (2′-C), 78.4 (3′-C), 71.4 (4′-C), 77.9 (5′-C), 62.7 (6′-C). ^1H NMR (400 MHz, CD_3OD-d_4): Part A**:** δ_H 5.94 (1H, brs, 1-H), 7.58 (1H, s, 3-H), 4.01 (1H, m, 5-H), 2.47

(1H, dd, *J*=14.7, 9.0 Hz, 6-H_A), 2.73 (1H, m, 6-H_B), 6.05 (1H, brq, *J*=7.1 Hz, 9-H), 1.75 (3H, dd, *J*=7.3, 1.5 Hz, 10-H), 3.76 (3H, s, 11-OMe), 4.83 (1H, d, *J*=8.1 Hz, 1′-H), 3.25-3.43 (4H, m, 2′,3′,4′,5′-H), 3.68 (1H, m, 6′-H_A), 3.88 (1H, br d, *J*=12.0Hz, 6′-H_B), 7.29 (2H, d, *J*=8.5 Hz, 2″-H), 3.42 (2H, t, *J*=9.5 Hz, 6″-H), 6.99 (2H, d, *J*=8.5 Hz, 3″,5″-H), 3.80 (1H, m, 7″-H_A), 4.03 (1H, dt, *J*=10.0, 7.3 Hz, 7″-H_B), 2.94 (2H, t, *J*=7.3 Hz, 8″-H), 4.31 (2H, d, *J*=7.8 Hz, 1‴-H), 3.19 (2H, br t, *J*=8.3 Hz, 2‴-H), 3.25-3.43 (1H, m, 4‴-H), 3.42 (1H, m, 5‴-H), 4.21 (1H, dd, *J*=12.0, 5.6 Hz, 7‴-H), 4.34 (1H, dd, *J*=12.0,2.0Hz, 8‴-H); Part B: δ_H 6.04 (1H, br s, 1-H), 7.52 (1H, s, 3-H), 4.11 (1H, dd, *J*=9.3, 4.4 Hz, 5-H), 2.73 (1H, dd, *J*=14.6, 9.3 Hz, 6-H_A), 2.96 (1H, dd, *J*=14.6, 4.4 Hz, 6-H_B), 6.18 (1H, br q, *J*=7.1 Hz, 9-H), 1.76 (3H, dd, *J*=7.3, 1.5 Hz, 10-H), 3.68 (3H, s, 11-OMe), 4.83 (1H, d, *J*=7.6 Hz, 1′-H), 3.25-3.43 (4H, m, 2′,3′,4′,5′-H), 3.66 (1H, m, 6′-H_A), 3.81 (1H, m, 6′-H_B).

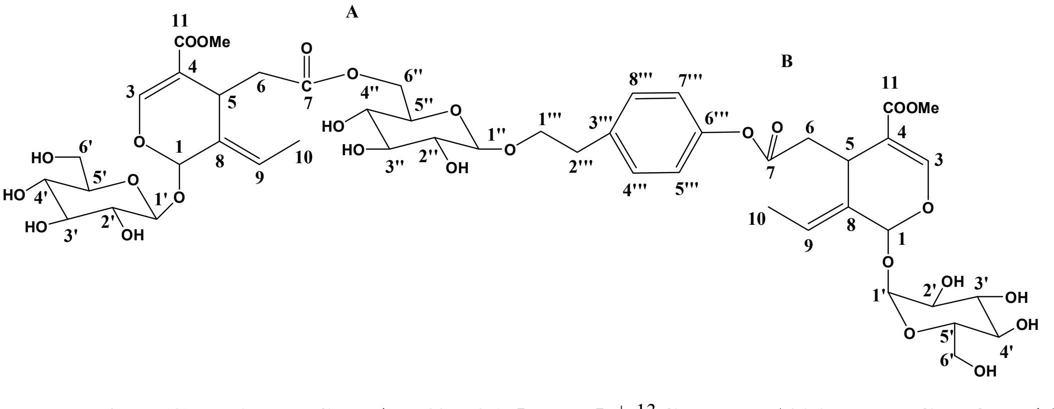

Nuzhenide (3): FAB-MS, m/z: 687.21 $[M+H]^+$. ^{13}C NMR (400MHz, CD_3OD-d_4): δ_C 95.2 (1-C), 155.2 (3-C), 109.4 (4-C), 31.8 (5-C), 41.3 (6-C), 173.1 (7-C), 130.7 (8-C), 125.0 (9-C), 13.8 (10-C), 168.8 (11-C), 52.1 (11-OMe), 100.8 (1′-C), 74.7 (2′-C), 78.4 (3′-C), 71.6 (4′-C), 77.9 (5′-C), 62.7 (6′-C), 104.5 (1″-C), 75.0 (2″-C), 77.9 (3″-C), 71.5 (4″-C), 75.1 (5″-C), 65.0 (6″-C), 130.4 (1‴-C), 131.0 (2‴, 6‴-C), 116.2 (3‴, 5‴-C), 156.7 (4‴-C), 36.4 (7‴-C), 72.3 (8‴-C). ^1H NMR (400 MHz, CD_3OD-d_4): δ_H

5.86 (1H, s, 1-H), 7.46 (1H, s, 3-H), 3.96 (1H, dd, *J*=8.5, 4.8 Hz, 5-H), 2.46 (1H, dd, *J*=14.0, 8.8 Hz, 6-H_A), 2.69 (1H, dd, *J*=14.2, 5.0 Hz, 6-H_B), 6.02 (1H, dd, *J*=6.8, 1.0 Hz, 9-H), 1.68 (3H, d, *J*=7.2 Hz, 10-H), 4.26 (1H, d, *J*=7.6 Hz, 1′-H), 3.28 (1H, dd, *J*=8.8, 7.8 Hz, 2′-H), 3.40 (1H, m, 3′-H), 3.26 (1H, m, 4′-H), 3.40 (1H, m, H-5′), 4.59 (1H, dd, *J*=12.0, 2.4 Hz, 6′-H_A), 4.18 (1H, dd, *J*=12.0, 5.6 Hz, 6′-H_B), 4.78 (1H, d, *J*=7.8 Hz, 1″-H), 3.22 (1H, dd, *J*=7.8, 8.8 Hz, 2″-H), 3.27 (3H, m, 3″, 4″, 5″-H), 3.92 (1H, dd, *J*=12.0, 2.4 Hz, 6″-H_A), 3.66 (1H, dd, *J*=12.0, 5.6 Hz, 6″-H_B), 3.63 (3H, s, 11-OMe), 6.62 (1H, d, *J*=8.6 Hz, 2‴, 6‴-H), 6.99 (2H, d, *J*=8.6 Hz, 3‴, 5‴-H), 2.75 (2H, m, 7‴-H), 3.91 (1H, m, 8‴-H_A), 3.60 (1H, dt, *J*=8.4 Hz, 8‴-H_B).

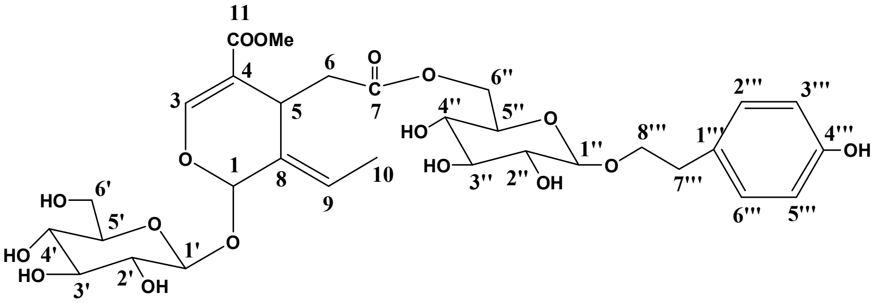

Ligstroside (4): FAB-MS, m/z: 524.98 $[M+H]^+$. ^{13}C NMR (400MHz, CD_3OD-d_4): δ_C 95.2 (1-C), 155.3 (3-C), 109.5 (4-C), 31.9 (5-C), 41.4 (6-C), 173.3 (7-C), 130.2 (8-C), 125.0 (9-C), 13.7 (10-C), 168.8 (11-C), 52.1 (11-OMe), 101.0 (1′-C), 74.9 (2′-C), 78.6 (3′-C), 71.6 (4′-C), 78.1 (5′-C), 62.8 (6′-C), 67.1 (1″-C), 35.3 (2″-C), 130.6 (3″-C), 131.2 (4″-C), 116.4 (5″-C), 157.2 (6″-C) ,116.4 (7″-C), 131.2 (8″-C). ^1H NMR (400 MHz, CD_3OD-d_4): δ_H 5.89 (1H, s, 1-H), 7.50 (1H, s, 3-H), 4.00 (1H, dd, *J*=9.2, 4.4 Hz, 5-H), 2.69 (1H, dd, *J*=14.0, 4.4 Hz, 6-H_A), 2.45 (1H , dd, *J*=14.0, 9.6 Hz, 6-H_B), 6.05 (1H, q, *J*=7.2 Hz, 9-H), 1.61 (3H, d, *J*=7.2 Hz, 10-H), 4.78 (1H, d, *J*=8.0 Hz, 1′-H), 3.38 (2H, m, 2′,4′-H), 3.61 (1H, m, 3′-H), 3.31 (1H, m, 5′-H), 3.89 (1H, dd, *J*=12.0,

2.4 Hz, 6′-H_A), 3.63 (1H, dd, *J*=12.0, 5.6 Hz, 6′-H_B), 3.70 (3H, s, 11-OMe),4.20 (1H, m, 1″-H_A), 4.08 (1H, m, 1″-H_B), 2.80 (2H, t, *J*=6.8 Hz, 2″-H), 7.00 (2H, d, *J*=8.4 Hz, 4″, 8″-H), 6.69 (2H, d, *J*=8.4 Hz, 5″, 7″-H).

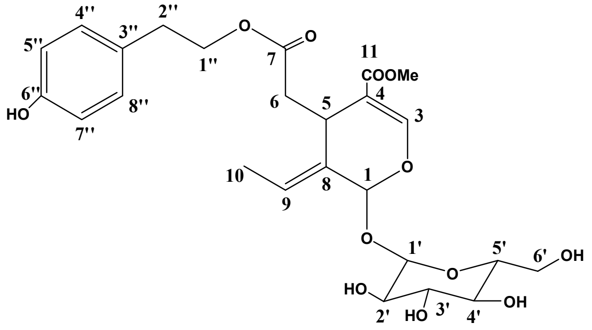

Oleoside-11-methyl ester (5): FAB-MS, m/z: 405.16 $[M+H]^+$. ^{13}C NMR (400 MHz, CD_3OD-d_4): δ_C 93.6 (1-C), 153.7 (3-C), 107.9 (4-C), 30.4 (5-C), 39.6 (6-C), 172.1 (7-C), 129.0 (8-C), 123.4 (9-C), 12.2 (10-C), 167.2 (11-C), 50.8 (11-OMe), 99.4 (1′-C), 73.3 (2′-C), 77.0 (3′-C), 70.0 (4′-C), 76.5 (5′-C), 61.2 (6′-C). ^1H NMR (400 MHz, CD_3OD-d_4): δ_H 5.94 (1H, s, 1-H), 7.54 (1H, s, 3-H), 4.03 (1H, dd, *J*=9.2, 4.4 Hz, 5-H), 2.79 (1H, dd, *J*=14.0, 4.4 Hz, 6-H_A), 2.50 (1H, dd, *J*=14.0, 9.6 Hz, 6-H_B), 6.16 (1H, dd, *J*=7.2, 6.6 Hz, 9-H), 1.76 (3H, d, *J*=7.2 Hz, 10-H), 4.83 (1H, d, *J*=8 Hz, 1′-H), 3.38 (1H, m, 2′-H), 3.41 (1H, m, 3′-H), 3.46 (1H, m, 4′-H), 3.33 (1H, m, 5′-H), 3.92 (1H, brd, *J*=11.6 Hz, 6′-H_A), 3.71 (1H, m, 6′-H_B), 3.74 (3H, s, 11-OMe).

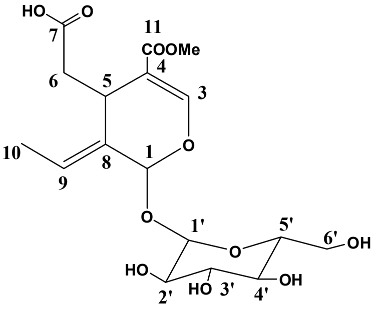

Oleuricine A (6): FAB-MS, m/z: 702.93 $[M+H]^+$. ^{13}C NMR (400 MHz, DMSO-d_6): δ_C 92.7(1-C), 153.3 (3-C), 107.6 (4-C), 30.0 (5-C), 40.0 (6-C), 170.5 (7-C), 129.1

(8-C), 123.3 (9-C), 13.0 (10-C), 166.1 (11-C), 51.1 (11-OMe), 98.8 (1′-C), 73.4 (2′-C), 76.3 (3′-C), 70.0 (4′-C), 73.1 (5′-C), 69.8 (6′-C), 102.7 (1″-C), 73.1 (2″-C), 76.4 (3″-C), 70.0 (4″-C), 77.2 (5″-C), 128.5 (1‴-C), 115.0 (2‴-C), 129.6 (3‴-C), 155.5 (4‴-C),129.6 (5‴-C), 115.0 (6‴-C), 64.0 (7‴-C), 34.7 (8‴-C). ^{1}H NMR (400 MHz, DMSO-d$_6$): δ$_H$ 5.86 (1H, s, H-1), 7.49 (1H, s, H-3), 3.84 (1H, dd, *J*=8.0, 4.0 Hz, H-5), 2.38 (1H, m, H-6$_A$), 2.74 (1H, m, H-6$_B$), 5.95 (1H, m, H-9), 1.67 (3H, d, *J*= 6.0 Hz, H-10), 3.59 (3H, s, 11-OMe), 4.62 (1H, d, *J*=8.0 Hz, H-1′), 2.98-3.36 (7H, m, H-2′, 3′, 4′, 5′, 3″, 4″, 5″), 3.55 (2H, m, H-6′), 4.20 (1H, d, *J*=7.6 Hz, H-1″), 3.36 (1H, m H-2″), 4.19 (2H, m, H-6″), 6.45 (2H, d, *J*=8.0 Hz, H-2‴,6‴), 6.60 (2H, d, *J*=8.0 Hz, H-3‴,5‴), 4.24 (1H, m, H$_A$-7‴), 4.00 (1H, m, H$_B$-7‴), 2.65 (2H, m, H-8‴).

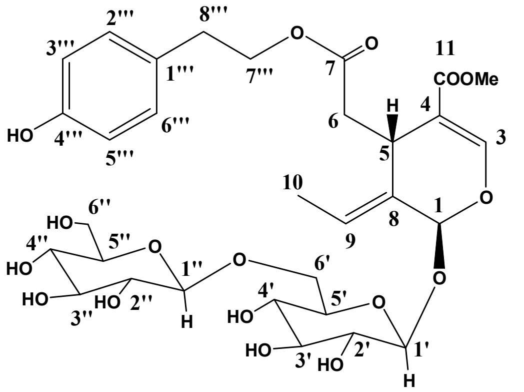

Nicotiflorine (7): FAB-MS, m/z: 688.06 [M+H]$^{+}$. ^{13}C NMR (400 MHz, DMSO-d$_6$): δ$_C$ 93.1 (1-C), 153.6 (3-C), 107.6 (4-C), 30.3 (5-C), 40.1 (6-C), 170.8 (7-C), 129.2 (8-C), 123.3 (9-C), 13.0 (10-C), 166.4 (11-C), 51.4 (11-OMe), 99.3 (1′-C), 73.4 (2′-C), 76.6 (3′-C), 70.0 (4′-C), 77.4 (5′-C), 61.2 (6′-C), 102.6 (1″-C), 73.4 (2″-C), 76.5 (3″-C), 70.1 (4″-C), 76.6 (5″-C), 60.8 (6″-C), 135.7 (1‴-C), 129.3 (2‴-C), 121.7 (3‴-C), 149.0 (4‴-C), 121.7 (5‴-C), 129.3 (6‴-C), 70.1 (7‴-C), 33.7 (8‴-C). ^{1}H NMR (400 MHz, CD$_3$OD-d$_4$): δ$_H$ 6.02 (1H, brs, H-1), 7.58 (1H, s, H-3), 4.10 (1H, dd,

J=9.5, 4.5 Hz, H-5), 2.74 (1H, dd, *J*=15.0, 9.5 Hz, H_A-6), 2.95 (1H, dd, *J*=15.0, 4.5 Hz, H_B-6), 6.19 (1H, dq, *J*=7.0, 1.2 Hz, H-9), 1.77 (3H, dd, *J*=7.0, 1.5 Hz, H-10), 3.74 (3H, s, 11-OMe), 3.83 (1H, dd, *J*=12.0, 1.2 Hz, H_B-6′), 3.62 (1H, dd, *J*=12.0, 5.5 Hz, H_A-6′), 3.40 (1H, t, *J*=9.0 Hz, H-3′), 4.79 (1H, d, *J*=8.0 Hz, H-1′), 4.29 (1H, d, *J*=8.0 Hz, H-1″), 3.67 (1H, dd, *J*=12.0, 5.5 Hz, H_A-6″), 3.88 (1H, dd, *J*=12.0, 1.8 Hz, H_B-6″), 7.29 (2H, d, *J*=8.5 Hz, H-2‴, 6‴), 7.00 (2H, d, *J*=8.5 Hz, H-3‴, 5‴), 3.78 (1H, dt, *J*=10.0, 7.0 Hz, H_A-7‴), 4.09 (1H, dt, *J*=10.0, 7.0 Hz, H_B-7‴), 2.93 (2H, t, *J*=7.0 Hz, H-8‴).

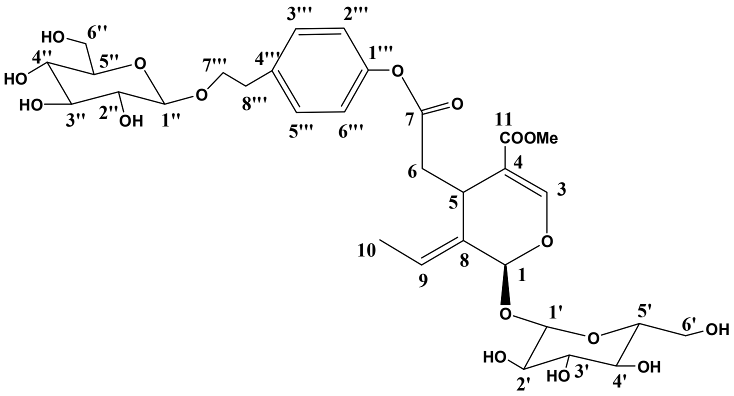

Jaspolyanoside (8): FAB-MS, m/z: 910.89 $[M+H]^+$. ^{13}C NMR (400 MHz, CD_3OD-d_4): Part A δ_C 95.5 (1-C), 155.3 (3-C), 109.2 (4-C), 31.7 (5-C), 41.1 (6-C), 173.5 (7-C), 130.6 (8-C), 124.8 (9-C), 13.7 (10-C), 168.5 (11-C), 51.8 (11-OMe), 100.7 (1′-C), 74.6 (2′-C), 77.8 (3′-C), 71.3 (4′-C), 75.0 (5′-C), 64.2 (6′-C), 130.3 (1″-C), 130.8 (2″-C), 116.2 (3″-C), 156.6 (4″-C), 116.0 (5″-C), 130.8 (6″-C), 72.1 (7″-C), 36.2 (8″-C); Part B δ_C 95.2 (1-C), 155.3 (3-C), 109.5 (4-C), 31.7 (5-C), 41.2 (6-C), 173.8 (7-C), 130.3 (8-C), 124.8 (9-C), 13.4 (10-C), 168.4 (11-C), 52.3 (11-OMe), 100.6 (1′-C), 74.6 (2′-C), 78.2 (3′-C), 71.5 (4′-C), 78.3 (5′-C), 62.7 (6′-C). ^1H NMR (400 MHz, CD_3OD-d_4): Part A: δ_H 5.91 (1H, q, *J*=1.6 Hz, H-1), 7.50 (1H, m,

H-3), 3.89 (1H, m, H-5), 2.21 (1H, m, H_A-6), 2.76 (1H, m, H_B-6), 6.09 (1H, dtd, *J*=18.6, 7.6, 6.2 Hz, H-9), 1.63 (3H, m, H-10), 3.67 (3H, m, OMe-11), 4.79 (1H, dd, *J*=7.8, 3.3 Hz, H-1′), 3.49–3.09 (4H, m, H-2′, 3′, 4′, 5′), 4.29 (2H, m, H-6′), 7.04 (2H, m, H-2″,6″), 6.69 (2H, m, H-3″, 5″), 4.01 (1H, m, H_A-7″), 4.30 (1H, m, H_B-7″), 2.76 (2H, m, H-8″); Part B δ_H 6.00 (1H, t, *J*=1.7 Hz, H-1), 7.50 (1H, m, H-3), 4.01 (1H, m, H-5), 2.21 (1H, m, H_A-6), 2.76 (1H, m, H_B-6), 6.09 (1H, dd, *J*=18.6, 6.2 Hz, H-9), 1.63 (3H, m, H-10), 3.70 (3H, s, OMe-11), 4.79 (2H, dd, *J*=7.8, 3.3 Hz, H-1′), 3.49–3.09 (4H, m, 2′, 3′, 4′, 5′-H), 3.67 (1H, m, H_A-6′), 3.89 (1H, m, H_B-6′).

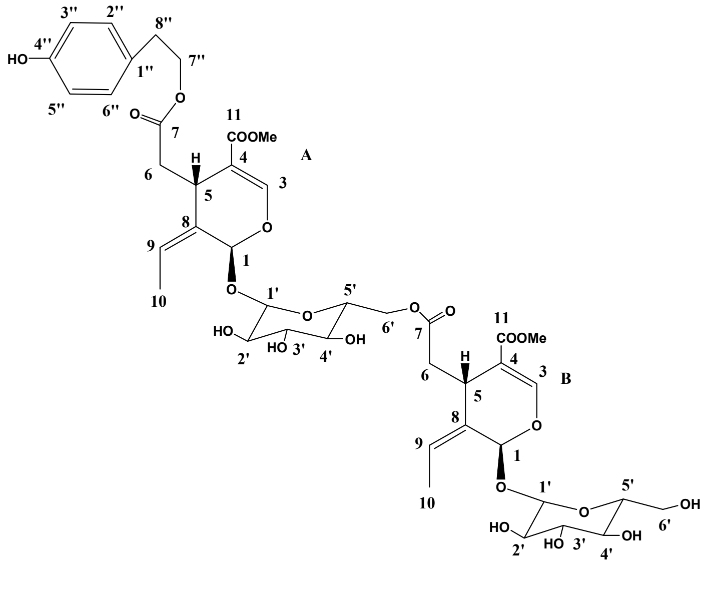

Oleopolynuzhenide A (9): FAB-MS, m/z: 1459.20 [M+H] $^+$. ^{13}C NMR (400 MHz, CD_3OD-d_4): Part A δ_C 95.2 (1-C), 155.4 (3-C), 109.5 (4-C), 31.9 (5-C), 41.3 (6-C), 173.7 (7-C), 130.7 (8-C), 125.2 (9-C), 13.9 (10-C), 168.8 (11-C), 52.3 (11-OMe), 101.1 (1′-C), 74.9 (2′-C), 78.5 (3′-C), 71.6 (4′-C), 78.0 (5′-C), 62.8 (6′-C), 137.3 (1″-C), 131.2 (2″-C), 122.8 (3″-C), 150.9 (4″-C), 122.8 (5″-C), 131.2 (6″-C), 71.6 (7″-C), 35.4 (8″-C), 101.2 (1‴-C), 74.9 (2‴-C), 75.7 (3‴-C), 71.5 (4‴-C), 75.1 (5‴-C), 62.7 (6‴-C); Part B δ_C 95.2 (1-C), 155.2 (3-C), 109.4 (4-C), 31.9 (5-C), 41.3

(6-C), 171.7 (7-C), 130.6 (8-C), 125.0 (9-C), 13.6 (10-C), 168.7 (11-C), 52.1 (11-OMe), 101.0 (1′-C), 74.9 (2′-C), 78.5 (3′-C), 71.5 (4′-C), 78.0 (5′-C), 62.8 (6′-C); Part C δ_C 95.4 (1-C), 155.2 (3-C), 109.4 (4-C), 31.9 (5-C), 41.1 (6-C), 173.3 (7-C), 130.4 (8-C), 124.9 (9-C), 13.7 (10-C), 168.8 (11-C), 52.0 (11-OMe), 100.9 (1′-C), 74.9 (2′-C), 78.5 (3′-C), 71.5 (4′-C), 78.0 (5′-C), 62.9 (6′-C). ^{1}H NMR (400 MHz, CD_3OD-d_4): Part A δ_H 5.91 (1H, q, *J*=1.5 Hz, H-1), 7.53 (1H, m, H-3), 4.00 (1H, m, H-5), 2.43 (1H, m, H_A-6), 2.74 (1H, m, H_B-6), 6.10 (1H, m, H-9), 1.76 (3H, ddd, *J*=8.8, 7.1, 1.5 Hz, H-10), 3.69 (3H, m, OMe-11), 4.81 (1H, dd, *J*=10.1, 7.7 Hz, H-1′), 3.43–3.25 (4H, m, H-2′, 3′, 4′, 5′), 3.69 (1H, m, H_A-6′), 3.81 (1H, d, *J*=1.4 Hz, H_B-6′), 7.30 (2H, m, H-2″, 6″), 7.04 (2H, t, *J*=8.1 Hz, H-3″, 5″), 3.71 (1H, m, H_A-7″), 3.98 (1H, m, H_B-7″), 2.96 (2H, m, H-8″), 4.83 (1H, dd, *J*=10.1, 7.7 Hz, H-1‴), 4.20 (1H, m, H-2‴), 3.43-3.25 (3H, m, H-3‴, 4‴, 5‴), 3.69 (1H, m, H_A-6‴), 3.81 (1H, d, *J*=1.4 Hz, H_B-6‴); Part B δ_H 5.91 (1H, d, *J*=1.5 Hz, H-1), 7.53 (1H, m, H-3), 4.20 (1H, m, H-5), 2.76 (1H, m, H_A-6), 2.94 (1H, m, H_B-6), 6.10 (1H, m, H-9), 1.76 (3H, ddd, *J*= 8.8, 7.1, 1.5 Hz, H-10), 3.69 (3H, m, OMe-11), 4.81 (1H, dd, *J*=10.1, 7.7 Hz, H-1′), 3.43–3.25 (4H, m, H-2′, 3′, 4′, 5′), 3.69 (1H, m, H_A-6′), 3.81 (1H, d, *J*=1.4 Hz, H_B-6′); Part C δ_H 5.91 (1H, q, *J*=1.5 Hz, H-1), 7.53 (1H, m, H-3), 3.98 (1H, m, H-5), 2.46 (1H, m, H_A-6), 2.74 (1H, m, H_B-6), 6.10 (1H, m, H-8), 1.76 (3H, ddd, *J*=8.8, 7.1, 1.5 Hz, H-10), 3.69 (3H, m, OMe-11), 4.81 (1H, dd, *J*=10.1, 7.7 Hz, H-1′), 3.43–3.25 (4H, m, H-2′, 3′, 4′, 5′), 3.69 (1H, m, 6′-H_A), 3.81 (1H, d, *J*=1.4 Hz, H_B-6′).

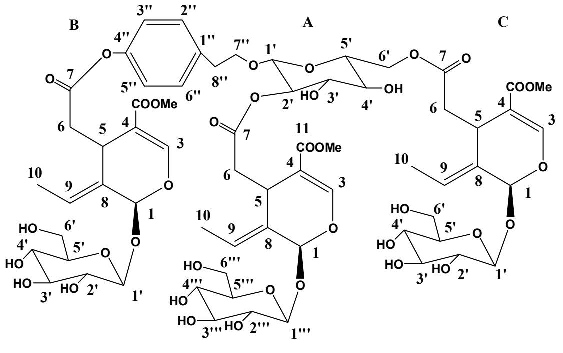

Safghanoside G (10): FAB-MS, m/z: 1015.85 $[M+H]^+$. ^{13}C NMR (400 MHz, CH_3OD-d_4): Part A δ_C 95.2 (1-C), 155.4 (3-C), 109.5 (4-C), 31.9 (5-C), 41.3 (6-C), 173.6 (7-C), 130.5 (8-C), 125 (9-C), 13.7 (10-C), 168.7 (11-C), 101.1 (1′-C), 74.8 (2′-C), 78 (3-C′), 71.6 (4′-C), 78.5 (5′-C), 62.8 (6′-C), 130.1 (1″-C), 131.1 (2″-C), 116.4 (3″-C), 156.9 (4″-C), 116.4 (5″-C), 131.0 (6″-C), 65.1 (7″-C), 36.5 (8″-C); Part B δ_C 95.4 (1-C), 155.3 (3-C), 109.4 (4-C), 31.9 (5-C), 41.2 (6-C), 171.8 (7-C), 130.7 (8-C), 125.2 (9-C), 14.0 (10-C), 168.8 (11-C), 52.1 (11-OMe), 100.9 (1′-C), 75.0 (2′-C), 78.0 (3′-C), 71.7 (4′-C), 78.5 (5′-C), 62.8 (6′-C), 138 (1″-C), 131.1 (2″-C), 122.6 (3″-C), 150.6 (4″-C), 122.6 (5″-C), 131.0 (6″-C), 65.1 (7″-C), 36.7 (8″-C). ^1H NMR (400 MHz, CD_3OD-d_4): Part A δ_H 5.91 (1H, d, *J*=1.7 Hz, H-1), 7.51 (2H, d, *J*=2.7 Hz, H-3), 4.00 (1H, m, H-5), 2.49 (1H, m, H_A-6), 2.78 (1H, m, H_B-6), 6.05 (1H, m, H-9), 1.73 (3H, m, H-10), 4.81 (1H, m, H-1′), 3.53–3.30 (4H, m, H-2′, 3′, 4′, 5′), 3.69 (1H, m, H_A-6′), 3.84 (1H, m, H_B-6′), 7.00 (2H, m, H-2″, 6″), 6.69 (2H, m, H-3″, 5″), 4.10 (1H, m , H_A-7″), 4.30 (1H, m, H_B-7″), 2.78 (2H, m, H-8″); Part B δ_H 5.91 (1H, d, *J*=1.7 Hz, H-1), 7.50 (1H, d, *J*=2.7 Hz, H-3), 4.00 (1H, m, H-5), 2.78 (1H, m, H_A-6), 2.95 (1H, m, H_B-6), 6.14 (1H, m, H-9), 1.75 (3H, m, H-10), 3.69 (3H, m, OMe-11), 4.81 (1H, m, H-1′), 3.30 (4H, m, H-2′, 3′, 4′, 5′), 3.69 (1H, m, H_A-6′), 3.86 (1H, m, H_B-6′), 7.29 (2H, m, H-2″,6″), 7.00 (2H, m, H-3″, 5″), 4.30 (1H, m, H_A-7″),

4.29 (1H, m, H_B-7″), 2.95 (2H, m, H-8″).

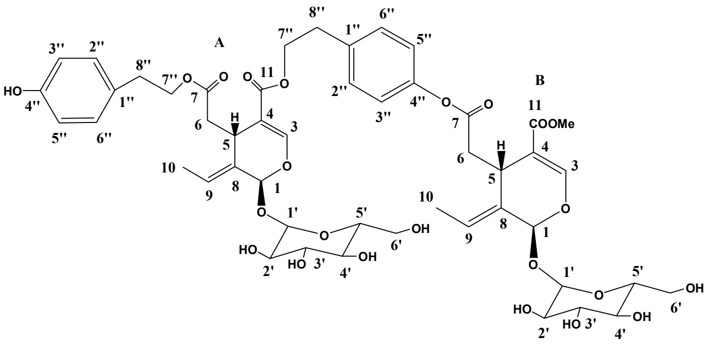

Excelside B (11): FAB-MS, m/z: 688.05 $[M+H]^+$. ^{13}C NMR (400 MHz, CH_3OD-d_4): δ_C 95.3 (1-C), 155.3 (3-C), 109.6 (4-C), 31.9 (5-C), 41.5 (6-C), 173.2 (7-C), 130.7 (8-C), 125.1 (9-C), 13.9 (10-C), 168.8 (11-C), 52.1 (11-OMe), 100.9 (1′-C), 78.2 (2′-C), 78.0 (3′-C), 71.7 (4′-C), 74.8 (5′-C), 70.0 (6′-C), 104.5 (1″-C), 75.2 (2″-C), 78.2 (3″-C), 71.6 (4″-C), 78.0 (5″-C), 62.8 (6″-C), 130.8 (1‴-C), 131.0 (2‴-C), 116.2 (3‴-C), 156.8 (4‴-C), 116.2 (5‴-C), 131.0 (6‴-C), 72.3 (7‴-C), 36.5 (8‴-C). ^{1}H NMR (400 MHz, CD_3OD-d_4): δ_H 5.93 (1H, m, H-1), 7.50 (1H, m, H-3), 3.93 (1H, m, H-5), 2.50 (2H, ddd, *J*=16.1, 14.3, 9.1 Hz, H-6), 6.10 (1H, dd, *J*=7.1, 1.7 Hz, H-9), 1.73 (3H, m, H-10), 3.70 (3H, m, OMe-11), 4.79 (1H, dd, *J*=7.8, 3.9 Hz, H-1′), 3.28–3.53 (7H, m, H-2′, 3′, 4′, 5′, 3″, 4″, 5″), 3.70 (2H, m, H_A-6′, 7‴), 4.10 (1H, m, H_B-6′), 4.30 (1H, m, H-1″), 3.19 (1H, m, H-2″), 3.83 (1H, dd, *J*=11.9, 5.8 Hz, H_A-6″), 3.63 (1H, m, H_B-6″), 7.05 (2H, dq, *J*=9.4, 3.1 Hz, H-2‴, 6‴), 6.70 (2H, m, H-3‴, 5‴), 3.95 (1H, m, H_B-7‴), 2.79 (2H, m, H-8‴).

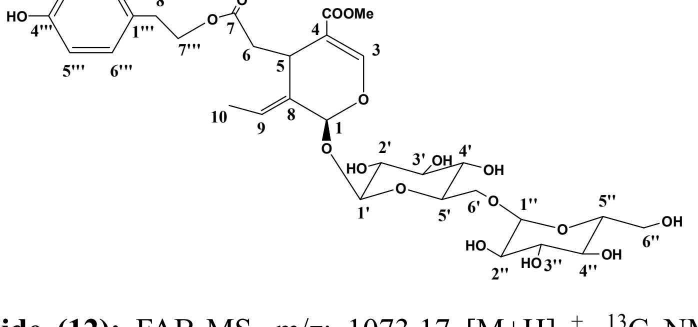

Isooleonuezhenide (12): FAB-MS, m/z: 1073.17 $[M+H]^+$. ^{13}C NMR (400 MHz, CH_3OD-d_4): δ_C 95.1 (1-C), 156.8 (3-C), 109.4 (4-C), 31.7 (5-C), 41.3 (6-C), 173.0 (7-C), 130.7 (8-C), 124.9 (9-C), 13.7 (10-C), 168.7 (11-C), 51.9 (11-OMe), 100.9

(1′-C), 74.8 (2′-C), 77.8 (3′-C), 71.5 (4′-C), 74.6 (5′-C), 65.1 (6′-C), 100.7 (1″-C), 74.8 (2″-C), 77.8 (3″-C), 71.7 (4″-C), 77.8 (5″-C), 62.5 (6″-C), 104.5 (1‴-C), 74.8 (2‴-C), 78.0 (3‴-C), 71.7 (4‴-C), 75.2 (5‴-C), 65.1 (6‴-C), 131.0 (1⁗-C), 130.0 (2⁗-C), 116.2 (3⁗-C), 155.3 (4⁗-C), 116.2 (5⁗-C), 130.0 (6⁗-C), 72.3 (7⁗-C), 36.5 (8⁗-C). ^{1}H NMR (400 MHz, CH_3OD-d_4): δ_H 5.91 (1H, t, *J*=1.7 Hz, H-1), 7.51 (1H, s, H-3), 3.96–4.00 (1H, m, H-5), 2.79 (1H, m, H_A-6), 2.50 (1H, dd, *J*=14.3, 8.8 Hz, H_B-6), 6.10 (1H, qd, *J*=7.2, 1.4 Hz, H-9), 1.74 (3H, s, H-10), 3.70 (3H, s, OMe-11), 4.89 (1H, d, *J*=7.5 Hz, H-1′), 3.23–3.54 (4H, m, H-2′, 3′, 4′, 5′), 4.31 (2H, m, H-6′), 4.31 (1H, m, H-1″), 3.5–3.2 (4H, m, H-2″,3″,4″,5″), 4.3 (2H, m, H-6″), 4.3 (1H, m, H-1‴), 3.23–3.54 (4H, m, H-2‴,3‴,4‴,5‴), 4.31 (1H, m, 6-H‴), 7.07 (2H, m, H-2⁗,6⁗), 6.70 (2H, m, H-3⁗,5⁗), 3.70 (1H, s, H_A-7⁗), 3.94 (1H, m, H_B-7⁗), 2.79 (2H, m, H-8⁗).

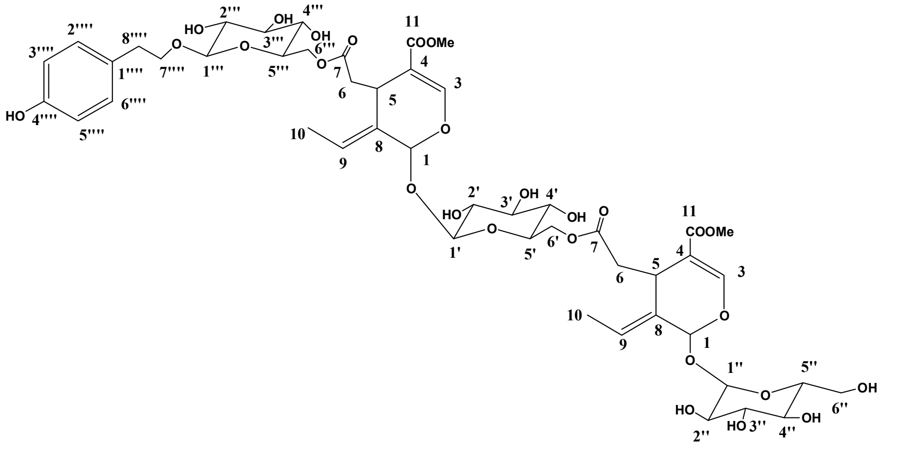

Lucidumoside A (13): FAB-MS, m/z: 528.30 [M+H] $^{+}$.^{13}C NMR (400 MHz, CD_3OD-d_4):δ_C 95.2 (1-C), 155.3 (3-C), 109.5 (4-C), 31.9 (5-C), 36.5 (6-C), 173.1 (7-C), 37.3 (8-C), 35.2 (9-C), 13.8 (10-C), 168.8 (11-C), 52.0 (11-OMe), 100.9 (1′-C), 74.8 (2′-C), 78.0(3′-C), 71.6 (4′-C), 78.5 (5′-C), 62.8 (6′-C), 130.1 (1″-C), 131.0 (2″-C), 116.5 (3″-C), 156.9 (4″-C), 116.2 (5″-C), 131.0 (6″-C), 65.1 (7″-C), 35.5

(8″-C). ^{1}H NMR (400 MHz, CD_3OD-d_4):δ_H 5.41 (1H, m, H-1), 7.50 (1H, m, H-3), 3.28 (1H, m, H-5), 2.49 (1H, dd, *J*=9.0, 13.2 Hz, H_A-6), 2.66 (1H, dd, *J*=3.1, 13.2 Hz, H_B-6), 1.78 (1H, m, H_A-9), 1.59 (1H, m, H_B-9), 2.09 (2H, m, H-8), 1.16 (3H, m, H-10), 3.70 (3H, m, OMe-11), 4.70 (1H, d, *J*=7.8 Hz, H-1′), 3.53–3.28 (4H, m, H-2′, 3′, 4′, 5′), 7.07 (2H, d, *J*=9.4 Hz, H-2″, 6″), 6.70 (2H, m, H-3″, 5″), 4.20 (2H, m, H-7″), 2.82 (2H, m, H-8″).

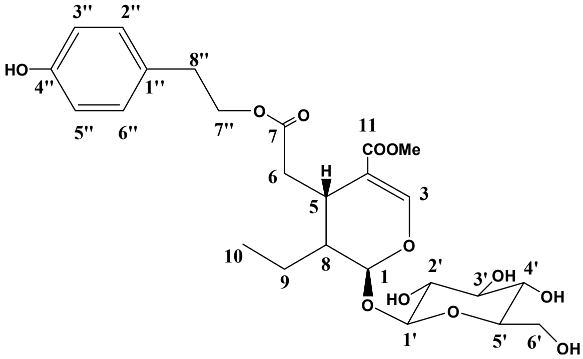

Safghanoside A (14): FAB-MS, m/z: 696.06 $[M+H]^+$. ^{13}C NMR (400 MHz, CD_3OD-d_4): δ_C 95.2 (1-C), 155.3 (3-C), 109.5 (4-C), 31.9 (5-C), 41.4 (6-C), 173.1 (7-C), 130.6 (8-C), 125.0 (9-C), 13.7 (10-C), 168.8 (11-C), 52.1 (11-OMe), 100.9 (1′-C), 74.8 (2′-C), 78.0 (3′-C), 71.6 (4′-C), 77.8 (5′-C), 62.8 (6′-C), 95.7 (1″-C), 75.1 (2″-C), 78.5 (3″-C), 71.7 (4″-C), 75.2 (5″-C), 65.1 (6″-C), 170.0 (1‴-C), 116.5 (2‴-C), 147.6 (3‴-C), 135.9 (4‴-C), 130.8 (5‴-C), 131.0 (6‴-C), 131.2 (7‴-C) ,131.0 (8‴-C), 130.8 (9‴-C). ^{1}H NMR (400 MHz, CD_3OD-d_4): δ_H 5.93 (1H, dt, *J*=8.9, 1.7 Hz, H-1), 7.56 (1H, m, H-3), 3.99 (1H, m, H-5), 2.50 (1H, ddd, *J*=7.4, 4.3, 9.2 Hz, H_A-6), 2.73 (1H, tt, *J* =9.2, 5.7 Hz, H_B-6), 6.09 (1H, m, H-9), 1.72 (3H, m, H-10), 3.70 (3H, s, OMe-11), 4.83 (1H, m, H-1′), 3.29–3.41 (8H, m, H-2′, 3′, 4′, 5′, 2″, 3″, 4″, 5″), 3.70 (1H, m, H_A-6′), 3.09 (1H, m, H_B-6′), 5.95 (1H, dt, *J*=8.9, 1.7 Hz, H-1″), 3.61 (2H, m, H-6″), 7.85 (2H, dq, *J*= 9.4 Hz, H-3‴), 7.62-7.40 (2H, m, H-5‴,

9‴).

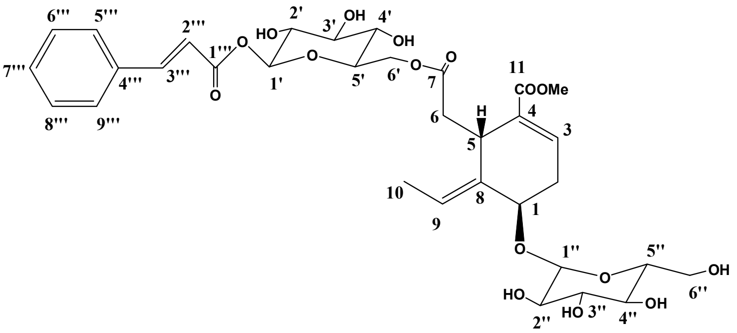

Jaspolyoleoside B (15): FAB-MS, m/z: 1295.93 [M+H] $^{+}$. ^{13}C NMR (400 MHz, CD_3OD-d_4): Part A δ_C 95.2 (1-C), 155.3 (3-C), 109.5 (4-C), 31.8 (5-C), 41.1 (6-C), 173.7 (7-C), 130.4 (8-C), 125.1 (9-C), 13.7 (10-C), 168.8 (11-C), 52.2 (11-OMe), 100.9 (1′-C), 74.7 (2′-C), 77.9 (3′-C), 71.5 (4′-C), 78.4 (5′-C), 62.7 (6′-C), 138.0 (1″-C), 131.1 (2″,6″-C), 122.5 (3″,5″-C), 150.5 (4″-C), 65.1 (7″-C), 36.4 (8″-C); Part B δ_C 95.4 (1-C), 155.4 (3-C), 109.3 (4-C), 31.9 (5-C), 41.4 (6-C), 171.8 (7-C), 130.7 (8-C), 125.3 (9-C), 13.9 (10-C), 168.8 (11-C), 52.2 (11-OMe), 100.5 (1′-C), 74.7 (2′-C), 77.9 (3′-C), 71.7 (4′-C), 75.1 (5′-C), 65.1 (6′-C); Part C δ_C 95.2 (1-C), 155.3 (3-C), 109.4 (4-C), 31.9 (5-C), 41.3 (6-C), 173.2 (7-C), 130.4 (8-C), 125.3 (9-C), 14.0 (10-C), 168.8 (11-C), 52.4 (11-OMe), 100.5 (1′-C), 75.0 (2′-C), 77.9 (3′-C), 71.7 (4′-C), 78.4 (5′-C), 62.7 (6′-C). ^{1}H NMR (400 MHz, CD_3OD-d_4): Part A δ_H 5.91 (1H, d, *J*=1.6 Hz, H-1), 7.56 (1H, m, H-3), 4.00 (1H, m, H-5), 2.50 (1H, ddt, *J*=6.2, 4.1, 6.3 Hz, H_A-6), 2.79 (1H, m, H_B-6), 6.03 (1H, d, *J*=1.7 Hz, H-9), 1.73 (3H, m, H-10), 3.69 (3H, m, OMe-11), 4.82 (1H, dd, *J*=7.7, 2.2 Hz, H-1′), 3.27–3.51 (4H, m, H-2′, 3′, 4′,5′), 3.69 (1H, m, H_A-6′), 3.83 (1H, m, H_B-6′), 7.29 (2H, m, H-2″,6″), 7.01 (2H, m, H-3″, 5″), 4.11 (1H, dd, *J*=9.1, 4.6 Hz, H_A-7″), 4.22 (1H, ddd, *J*=11.9, 5.8, 3.7 Hz, H_B-7″), 3.00 (2H, m, H-8″); Part B δ_H 5.92 (1H, s, H-1), 7.56 (1H, m, H-3), 4.22 (1H,

m, H-5), 2.79 (1H, m, H_A-6), 2.97 (1H, m, H_B-6), 6.18 (1H, m, H-9), 1.73 (3H, m, H-10), 3.69 (3H, m, OMe-11), 4.82 (1H, dd, *J*=7.7, 2.2 Hz, H-1′), 3.27-3.51 (3H, m, H-2′, 3′, 4′), 3.53 (1H, dd, *J*=9.5, 6.0 Hz, H-5′), 4.01 (1H, m, H_A-6′), 4.22 (1H, m, H_B-6′); Part C δ_H 7.56 (1H, m, H-3), 5.70 (1H, m, H-1), 3.99 (1H, m, H-5), 2.49 (1H, ddt, *J*=14.3, 14.1, 6.3 Hz, H_A-6), 2.79 (1H, m, H_B-6), 6.03 (2H, d, *J*=1.7 Hz, H-9), 1.73 (3H, m, H-10), 3.69 (3H, m, OMe-11), 4.82 (1H, dd, *J*=7.7, 2.2 Hz, H-1′), 3.27–3.51 (4H, m, H-2′,3′,4′,5′), 3.69 (1H, m, H_A-6′), 3.83 (1H, m, H_B-6′).

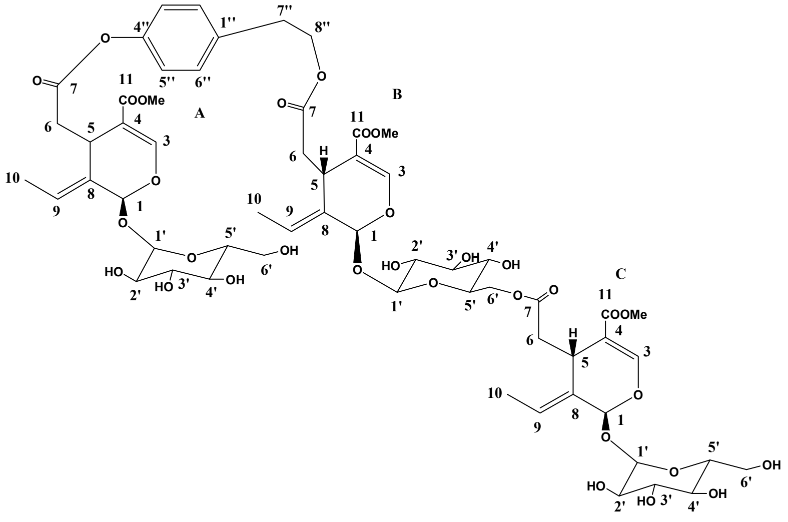

10-hydroxoleoside-7,11-dimethyl ester (16): FAB-MS, m/z: 435.23 $[M+H]^+$. ^{13}C NMR (400 MHz,, CD_3OD-d_4): δ_C 95.6 (1-C), 155.1(3-C), 109.2(4-C), 32.4 (5-C), 41.0 (6-C), 173.5 (7-C), 131.0 (8-C), 129.5 (9-C), 59.2 (10-C), 168.5 (11-C), 52.3 (11-OMe), 52.0 (7-OMe), 101.0 (1′-C), 78.0 (2′-C), 78.5 (3′-C), 71.5 (4′-C), 74.8 (5′-C), 62.7 (6′-C). ^1H NMR (400 MHz, CD_3OD-d_4): δ_H 5.91 (1H, s, 1-H), 7.49 (1H, s, 3-H), 4.26 (1H, dd, *J*=13.2, 7.2 Hz, 5-H), 2.69 (1H, dd, *J*=14.0, 4.4 Hz, 6-H_A), 2.44 (1H, dd, *J*=14.0, 9.6 Hz, 6-H_B), 6.09 (1H, q, *J*=7.2 Hz, 8-H), 3.89 (2H, d, *J*=8.8 Hz, 10-H), 4.15 (3H, d, *J*=4.8 Hz, 10-H, 4.80 (1H, d, *J*=8.2 Hz, 1′-H), 3.28 (1H, m, 2′-H), 3.38 (1H, m, 3′-H), 3.36 (1H, m, 4′-H), 3.30 (1H, m, 5′-H), 3.85 (1H, brd, *J*=11.4 Hz,

6′-H_A), 3.62 (1H, m, 6′-H_B), 3.70 (3H, s, 7-OMe), 3.69 (3H, s, 11-OMe).

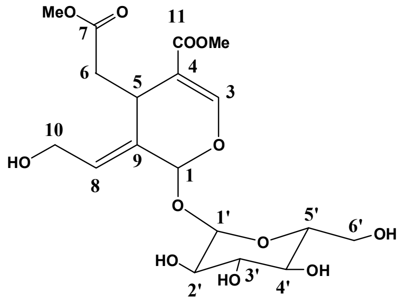

GI5 (29): FAB-MS, m/z: 910.24 [M+H] $^+$. ^{13}C NMR (400 MHz, CD_3OD-d_4): Part A δ_C 93.6 (1-C), 153.7 (3-C), 107.9 (4-C), 30.4 (5-C), 39.8 (6-C), 171.7 (7-C), 128.8 (8-C), 123.5 (9-C), 12.1 (10-C), 167.2 (11-C), 50.5 (11-OMe), 99.3 (1′-C), 73.3 (2′-C), 76.5 (3′-C), 70.0 (4′-C), 77.1 (5′-C), 61.3 (6′-C), 65.1 (1″-C), 33.9 (2″-C), 135.8 (3″-C), 129.6 (4″-C), 121.3 (5″-C), 149.3 (6″-C), 121.3 (7″-C), 131.2 (8″-C); Part B 93.8 (1-C), 153.9 (3-C), 107.8 (4-C), 30.3 (5-C), 39.6 (6-C), 170.2 (7-C), 129.1 (8-C), 123.7 (9-C), 12.4 (10-C), 167.2 (11-C), 50.6 (11-OMe), 99.5 (1′-C), 73.3 (2′-C), 76.5 (3′-C), 70.1 (4′-C), 77.0 (5′-C), 61.2 (6′-C). ^{1}H NMR (400 MHz, CD_3OD-d_4): δ_H 1.59 (3H, dd, *J*=6.9, 1.4 Hz, 10-H_A), 1.78 (3H, dd, *J*=6.9, 1.3 Hz, 10-H_B), 2.48 (1H, dd, *J*=12.0, 7.5 Hz, 6-H_A), 2.74 (1H, m, 6-H_A), 2.79 (1H, m, 6-H_B), 2.93 (2H, t, *J*=6.5 Hz, H-2″), 2.95 (1H, m, 6-H_B), 3.32-3.37 (8H, m, 2′$_A$, 3′$_A$, 4′$_A$, 5′$_A$, 2′$_B$, 3′$_B$, 4′$_B$, 5′-H), 3.69 (2H, m, 6′-H_A, 6′-H_B), 3.73 (3H, s, 11-OMe_A), 3.75 (3H, s, 11-OMe_B), 3.89 (1H, brd, *J*=14.0 Hz, 6′-H_A or H_B), 3.98 (1H, dd, *J*=10.0, 1.3 Hz, 6′-H_A or H_B), 4.16 (1H, dd, *J*=5.0, 2.5 Hz, 5-H_A), 4.19 (1H, dd, *J*=7.0, 2.5 Hz, 5-H_B), 4.35, 4.32 (each 1H, dt, *J*=6.0, 3.5 Hz, l″-H), 4.84 (1H, d, *J*=5.5 Hz, l′-H_A), 4.86 (1H, d, *J*=5.5 Hz, l′-H_B), 5.94 (1H, brs, l-H_A), 6.06 (1H, brs, l-H_B), 6.10 (1H, d, *J*=6.0 Hz, 9-H_A), 6.21 (1H, d, *J*=6.0 Hz, 9-H_B), 7.06 (2H, *J*=6.5 Hz, AA′BB′ pattern, 5″, 7″-H), 7.31 (2H, *J*=6.5 Hz,

AA′BB′ pattern, 4″, 8″-H), 7.53 (1H, s, 3-H_A), 7.59 (1H, s, 3-H_B).

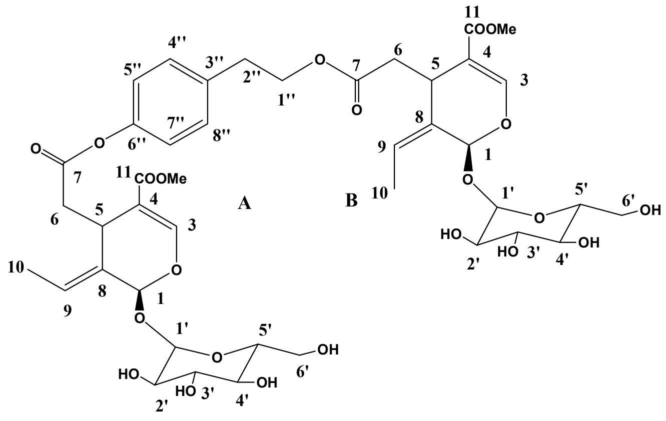

Oleoside-7,11-dimethyl ester (31): ESI-MS, m/z: 440.91 $[M+Na]^+$. ^{13}C NMR (400 MHz, CD_3OD-d_4): δ_C 93.6 (1-C), 153.7 (3-C), 107.9 (4-C), 30.4 (5-C), 39.6 (6-C), 172.1 (7-C), 129.0 (8-C), 123.4 (9-C), 12.2 (10-C), 167.2 (11-C), 50.5 (11-OMe), 50.8 (7-OMe), 99.1 (1′-C), 73.3 (2′-C), 76.4 (3′-C), 70.0 (4′-C), 77.0 (5′-C), 61.2 (6′-C). ^1H NMR (400 MHz, CD_3OD-d_4): δ_H 1.67 (3H, d, J=7.0 Hz, 10-H), 2.48 (1H, m, 6-H_A), 2.75 (1H, dd, J=4.3, 14.3 Hz, 6-H_B), 3.73 (6H, s, 7 and 11-OMe), 3.92 (1H, d, J=5.2 Hz, 5-H), 4.84 (1H, d, J=7.8 Hz, 1′-H), 5.94 (1 H, s, 1-H), 6.08 (1H, d, J=5.9 Hz, 9-H), 7.53 (1 H, s, 3-H), 3.34-3.97 (3H, m, 2′, 3′, 4′-H).

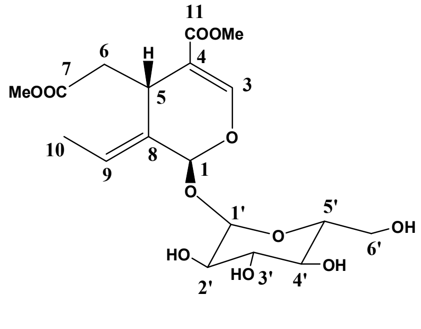

Isolignstroside (35): FAB-MS, m/z: 525.02 $[M+H]^+$. ^{13}C NMR (400 MHz, CD_3OD-d_4): δ_C 93.6 (1-C), 153.7 (3-C), 107.9 (4-C), 30.4 (5-C), 39.8 (6-C), 171.7 (7-C), 129.0 (8-C), 123.4 (9-C), 12.1 (10-C), 167.2 (11-C), 50.5 (11-OMe), 99.4 (1′-C), 73.3 (2′-C), 76.5 (3′-C), 70.0 (4′-C), 77.0 (5′-C), 61.3 (6′-C), 65.5 (1″-C), 33.7

(2″-C), 128.6 (3″-C), 129.6 (4″,8″-C), 114.8 (5″,7″-C), 155.6 (6″-C). ^{1}H NMR (400 MHz, CD_3OD-d_4): δ_H 5.94 (1H, s, H-1), 7.53 (1H, s, H-3), 4.00 (1H, dd, *J*=8.4, 5.2 Hz, H-5), 2.48 (1H, dd, *J*=10.8, 7.2 Hz, H_A-6), 2.75 (1H, dd, *J*=10.8, 6.2 Hz, H_B-6), 6.12 (1H, dd, *J*=8.8, 4.2 Hz, H-9), 1.67 (3H, d, *J*=7.8 Hz, H-10), 3.73 (3H, s, 7-OMe), 4.83 (1H, d, *J*=6.8 Hz, H-1′), 4.15-4.27 (2H, m, H-1″), 2.86 (2H, t, *J*=6.6, H-2″), 7.08 (2H, d, *J*=8.6, H-4″, 8″), 6.75 (2H, d, *J*=8.6, H-5″, 7″).

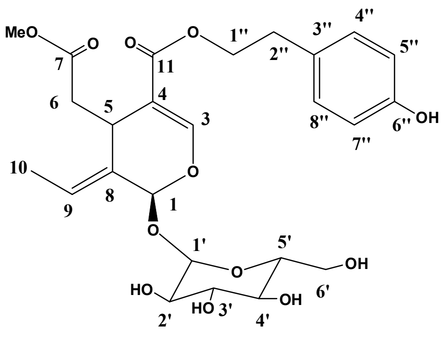

10-hydroxyligstroside (36): FAB-MS, m/z: 541.22 [M+H] $^{+}$. ^{13}C NMR (400 MHz, CD_3OD-d_4): δ_C 93.7 (1-C), 153.7 (3-C), 107.9 (4-C), 30.4 (5-C), 39.8 (6-C), 171.7 (7-C), 129.0 (8-C), 128.6 (9-C), 57.5 (10-C), 167.2 (11-C), 50.5 (11-OMe), 99.4 (1′-C), 73.3 (2′-C), 76.4 (3′-C), 70.0 (4′-C), 77.0 (5′-C), 61.3 (6′-C), 128.6 (1″-C), 114.9 (2″, 6″-C), 129.6 (3″, 5″-C), 155.6 (4″-C), 33.7 (7″-C), 65.5 (8″-C). ^{1}H NMR (400 MHz, CD_3OD-d_4): δ_H 2.47 (1H, dd, *J*=7.6, 12.0 Hz, 6-H_A), 2.75 (1H, dd, *J*=8.2, 12.0 Hz, 6-H_B), 2.83 (2H, t, *J*=6.7 Hz, 7″-H), 3.19 (1H, m, 5′-H), 3.34 (1H, m, 4′-H), 3.47 (1H, m, 3′-H), 3.72 (1H, m, 2′-H), 3.99 (1H, dd, *J*=6.1, 12.2 Hz, 6′-H_B), 3.37 (3H, s, 11-OMe), 3.93 (1H, d, *J*=10.8 Hz, 6′-H_A), 4.12 (1H, dd, *J*= 4.2, 9.0 Hz, 5-H), 4.24 (1H, dd, *J*=5.4, 12.6 Hz, 10-H_A), 4.25 (2H, m, 8″-H), 4.13 (1H, m, 10-H_B), 4.84 (1H, d, *J*=7.8 Hz, 1′-H), 5.93 (1H, s, 1-H), 6.10 (1H, dd, *J*=6.0, 4.4 Hz, 9-H), 6.75 (2H, d, *J*=8.4 Hz, 2″, 6″-H). 7.08 (2H, d, *J*=8.4 Hz, 3″, 5″-H), 7.53 (1H, s, 3-H).

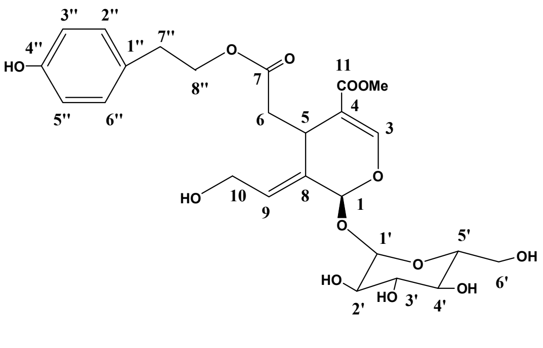
3''
2''
4''
1''
7''
HO
O
O
7
11
COOMe
8''
4
5''
6''
6
5
3
O
HO
10
8
1
9
O
1'
5'
O
OH
HO
HO
3'
OH
6'
2'
4'
